# Cooking-Derived Organic Compounds Shape Bacterial Communities on Household Surfaces

**DOI:** 10.1021/acs.est.5c02724

**Published:** 2025-07-07

**Authors:** Wing Lam Chan, Shicong Du, Huiju Lin, Yin Hau Lam, Jason Chun-Ho Lam, Theodora Nah, Patrick K. H. Lee

**Affiliations:** † School of Energy and Environment, 53025 City University of Hong Kong, Hong Kong SAR, China; ‡ State Key Laboratory of Marine Environmental Health, City University of Hong Kong, Hong Kong SAR, China; § School of Energy and Environment and State Key Laboratory of Marine Environmental Health, City University of Hong Kong, Hong Kong SAR, China; ∥ Low-Carbon and Climate Impact Research Centre, City University of Hong Kong, Hong Kong SAR, China

**Keywords:** indoor surfaces, bacterial communities, indoor chemicals, cooking

## Abstract

Understanding the factors influencing chemical and biological constituent accumulation on indoor surfaces is crucial, especially as individuals spend approximately 90% of their time indoors and frequently interact with these surfaces. However, the temporal relationships between these constituents remain unclear, as most field studies have relied on single-time snapshots and seldom examined the interplay between chemical and biological dynamics. We conducted a month-long spatiotemporal field study across 20 households in Hong Kong to investigate the factors influencing chemical and biological constituents on common indoor surfaces. Among the 16 household- and occupant-related factors analyzed, routine oil-based cooking was the primary driver of microbial diversity and composition on indoor surfaces. Surfaces in kitchens with frequent cooking exhibited elevated total organic carbon levels, which were linked to an increased bacterial abundance. A focused analysis of six kitchens with well-controlled frequencies of oil-based cooking revealed that cooking-derived organic compounds, particularly alkanes, promoted bacterial abundance while reducing microbial diversity. Network analysis further revealed strong interactions between these organic compounds and bacterial taxa, especially those within the *Proteobacteria* and *Firmicutes* phyla. These findings highlight the impact of routine household activities on indoor chemical–biological interactions, enhancing our understanding and informing strategies to improve indoor environments and occupant well-being.

## Graphical abstract

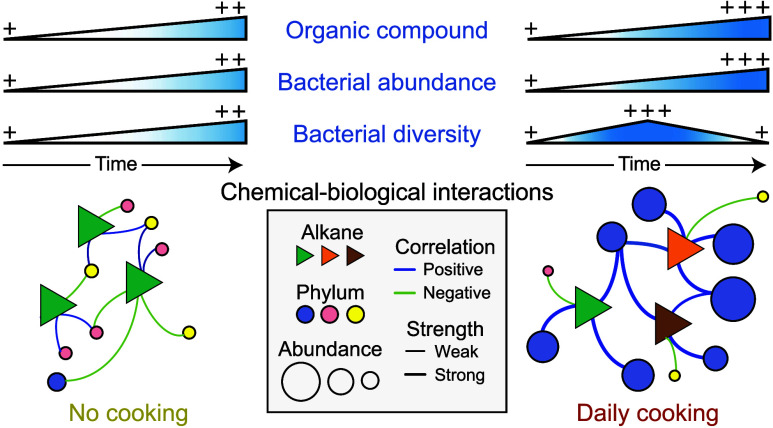

## Introduction

People spend ∼90% of their time indoors.^1^ Given their large surface-to-volume ratios, indoor surfaces can accumulate various chemical constituents, such as ions^2^ and organic carbons,^3^ and biological constituents, such as bacteria and fungi.^4^ This accumulation can significantly impact occupant health, potentially contributing to allergies, foodborne illnesses, and infectious diseases.^5,6^

Microbial and chemical constituent accumulation on indoor surfaces is influenced by various factors, including building design,^4,7^ occupant activities,^4,8^ direct physical contact,^9^ and airborne particle and compound deposition.^10,11^ Routine activities, such as food preparation, can increase surface bacterial concentrations,^4^ while organic compounds (*e.g.*, fatty acids, alkanes, and polycyclic aromatic hydrocarbons [PAHs]) released by cooking^12^ may influence bacterial growth and community dynamics. The routine application of chemical disinfectants to maintain surface hygiene can exert selective pressure, increasing disinfectant-resistant bacterial strains.^13^

Despite these insights, the interplay between indoor surface chemicals and bacterial communities remains unclear.^14^ Controlled laboratory studies have shown that specific indoor chemicals from cooking oil,^15^ human skin,^16^ and food^17,18^ can act as bactericidal agents or nutrients or have no discernible effect on different surface-associated bacterial species and strains. However, such experiments often involve high chemical concentrations and single model bacterial strains, which may not fully capture the diversity and complexity of real-world microbial communities in indoor environments. Furthermore, field studies have focused on isolated factors, such as geographical location^19^ and occupant density,^20^ that influence bacterial communities on household surfaces, neglecting the combined effects of multiple drivers. Many field studies have provided only snapshots by analyzing specific time points, missing the dynamic temporal variations in microbial communities.^21–24^ Thus, data on the complex temporal relationships between surface chemicals and microbial communities remain limited.

To address these knowledge gaps, we conducted a 1 month spatiotemporal field study across 20 households in Hong Kong to investigate the factors influencing bacterial communities on real-world household surfaces. Among the 16 household- and occupant-related factors analyzed, routine oil-based cooking emerged as a key contributor to high bacterial abundances, enhanced diversity, and altered composition *via* an increase in surface total organic carbon (TOC) concentrations. Further analysis of six kitchens with controlled oil-based cooking frequencies revealed that specific cooking-derived organic compounds exerted selective pressure, increasing bacterial abundances while reducing diversity and reshaping composition. This study highlights the significant yet often overlooked effect of routine household activities on chemical–biological interactions on indoor surfaces. A deeper understanding of these complex interactions can inform strategies to enhance indoor environments and occupant well-being.

## Materials and Methods

### Field Sampling and Sample Collection

Two field sampling rounds were conducted to investigate factors influencing bacterial communities on household surfaces: first, to identify key factors influencing these communities, and second, to understand their specific effects (Figure S1).

The first round, conducted over 30 consecutive days from late April to early June 2021, involved surface sampling in every room of 20 households across Hong Kong (Figure S2). Data on the following 16 factors influencing bacterial communities were obtained through measurements or a self-reported survey: three types of chemical constituents, ions, TOC, and total inorganic carbon (TIOC)—and 13 household—and occupant-related characteristics, namely, individual households (defined as all sampled rooms within a residential unit), geographical location, floor level, room type, room size, number of occupants, occupant density, oil-based cooking frequency, window-opening habits, cleaning routines, and use of air conditioners, cooling fans, and exhaust fans (Table S1). As oil-based cooking frequency in kitchens was identified as a key factor (see the Results section), samples were classified into three groups for downstream analyses: high-cooking-frequency (5–7 days/week), medium-cooking-frequency (3–4 days/week), and low-cooking-frequency kitchens (0–2 days/week), including nonkitchen rooms. All participating households were nonsmoking and pet-free. Temperature and relative humidity in each household’s living room were measured using a data logger (HOBO U12–013, Onset Computer Corporation, Bourne, MA, USA) (Table S1). To ensure uniformity, surface samples were collected using 30 soda-lime glass beads (3 mm diameter; total surface area = 8.5 cm^2^) arranged in a single layer within a polystyrene box (3.5 cm × 3.5 cm × 1 cm), following a previously described method^2^ with minor modifications for bead cleaning. Briefly, both the polystyrene boxes and glass beads were cleaned by soaking them in 5% Decon 90 solution (Decon Laboratories Ltd., Hove, U.K.) in an ultrasonic bath (40 kHz; 22 ± 2 °C) for 24 h.^25^ After rinsing and soaking in deionized water overnight, the boxes and beads were dried at 70 °C. Seven boxes containing glass beads, both open and closed, were deployed in each sampling room of the households. A closed-lid box served as a negative control for background subtraction in bacterial and chemical analyses, while six open-lid boxes were placed on tables ∼1 m above the ground and ∼2 m from windows in four room types: a bathroom, bedroom, kitchen, and living room. One box was collected from each room every 5 days for the analysis of water-soluble compounds using a TOC analyzer and ion chromatography (IC) system. Samples collected on days 5 and 30 were also analyzed for bacterial abundance and composition using quantitative polymerase chain reaction (qPCR) and 16S rRNA gene sequencing, respectively.

The second sampling round, conducted from October to November 2022, investigated the specific effects of oil-based cooking on bacterial communities in the kitchens of six selected households over a 30 day period (Figure S2). Households were chosen based on kitchen usage patterns observed in the first sampling round: three kitchens did not perform daily cooking, while the other three practiced daily oil-based cooking. To ensure consistency, certain activities (window opening, cleaning, and use of air conditioners, cooling fans, and exhaust fans) were prohibited, and strict adherence to each household’s cooking practices was enforced (Table S2). Samples were also classified into three groups for downstream analyses: (1) uncoated glass beads from daily cooking kitchens, representing exposure to both cooking emissions and associated environmental conditions; (2) uncoated glass beads from noncooking kitchens, providing a reference for environments without cooking influence; and (3) cooking-fume-coated glass beads placed in noncooking kitchens, isolating the effects of cooking-derived organics. This design enabled the controlled assessment of microbial responses to cooking-derived organic emissions versus broader environmental factors while accounting for initial differences in surface colonization. The uncoated glass beads were sampled following the same protocol as in the first round. One box was collected every 5 days, with a closed-lid box serving as a negative control. The samples were analyzed for chemical composition using a TOC analyzer and gas chromatography-mass spectrometry (GC-MS), while their bacterial communities were assessed using qPCR and 16S rRNA gene sequencing.

To assess the influence of cooking-derived organic compounds on bacterial communities on household surfaces, nine additional boxes containing glass beads coated with laboratory-generated cooking fumes were placed in each of the three noncooking kitchens. These beads were prepared by exposing 30 glass beads per box to fumes generated by heating 200 mL of 100% pure peanut oil (Knife, Hong Kong, China) at 200 °C for 20 min under ambient airflow. Glass beads from one box were analyzed immediately after coating using a TOC analyzer, GC-MS, qPCR, and 16S rRNA gene sequencing, serving as the baseline control for subsequent analyses. The remaining eight boxes—five with open lids and three with closed lids—were deployed in the noncooking kitchens. Closed-lid boxes served as controls to monitor changes in organic compounds due to abiotic factors. All boxes were collected every 10 days instead of every 5 days, as the uncoated beads, due to logistical constraints related to the recoating process. Glass beads from one open-lid and one closed-lid boxes were analyzed immediately using the same methods as those for the baseline control. The remaining boxes were recoated with cooking fumes to simulate continuous deposition. Subsequently, glass beads from one open-lid box were immediately analyzed to assess increases in organic compounds and changes in 16S rRNA gene copy numbers. The remaining recoated boxes were returned to the respective kitchens for continued temporal sampling. TOC concentrations on glass beads increased by 1.1 ± 0.4 μg/cm^2^ after each recoating procedure (paired Wilcoxon test, *p* < 0.05), compared with open-lid glass beads analyzed immediately after collection, indicating the effective deposition of organic compounds from cooking fumes during recoating (Figure S3). No bacteria were detected on closed-lid or baseline control glass beads. The 16S rRNA gene copy numbers on the glass beads tested immediately postrecoating showed no significant change relative to prerecoating levels at any collection time point (paired Wilcoxon test, *p* > 0.05), indicating minimal impact on bacteria already deposited on the beads prerecoated (Figure S3). TOC concentrations on coated or recoated beads from closed-lid boxes remained unchanged after 10 days (paired Wilcoxon test, *p* > 0.05; Figure S4), indicating minimal abiotic influence.

### Chemical Analysis

Chemical and biological constituents were extracted from glass beads by using sonication and filtration. TOC, TIOC, anions, and cations were quantified using TOC analysis and IC and organic compounds using GC-MS with internal standards following extraction, concentration, and filtration.^26^ Data processing involved peak integration and comparison against a standard chemical library. Detailed procedures are provided in Supporting Information Text S1.

### Genomic DNA Extraction, qPCR, and Amplicon Sequencing

Genomic DNA was extracted from glass beads, and the 16S rRNA gene was analyzed using qPCR, with absolute quantification performed using standard curves.^27^ 16S rRNA gene amplicon sequencing was conducted on an Illumina MiSeq platform, with appropriate positive and negative controls. Raw sequence data have been deposited in the NCBI Sequence Read Archive (accession number PRJNA1191072). Detailed procedures are provided in Supporting Information Text S2.

### Bioinformatics and Statistical Analyses

Bioinformatics analyses were performed using the QIIME2 platform^28^ for sequence processing, namely, quality filtering, denoising, amplicon sequence variant generation, and taxonomic assignment using the SILVA 138 database. Statistical analyses, including diversity metrics, differential abundance testing using the Analysis of Compositions of Microbiomes with Bias Correction (ANCOM-BC) algorithm, distance-based redundancy analysis (db-RDA), and co-occurrence network analysis, were performed in R using various packages. Bayesian generalized linear mixed-effects models, implemented using the Markov chain Monte Carlo estimation (MCMCglmm), were applied to evaluate the effects of factors on 16S rRNA gene copy numbers, α diversity (richness and Shannon index), and chemical concentration, with statistical significance assessed using Wald tests. Permutational multivariate analysis of variance (PERMANOVA) was used to assess differences in bacterial and organic compound compositions under different conditions. Co-occurrence network visualization and topological analysis were conducted using PyBootNet^29^ and Gephi. Detailed procedures are provided in Supporting Information Text S3, and the processed data and analysis code are publicly accessible on Zenodo (DOI: 10.5281/zenodo.15543432).

## Results

### Temporal Accumulation of Human-Associated Bacteria, Sodium and Chloride Ions, and Organic Carbon on Household Surfaces

Chemical and biological constituent accumulation on surfaces in four room types across 20 households was investigated over a 30 day period, measuring anion, cation, TOC, and TIOC concentrations and bacterial abundance and composition. Over the study period, the 16S rRNA gene copy number remained relatively stable across all tested household surfaces, ranging from 2.8 ± 1.5 to 3.2 ± 1.5 log_10_ copies/cm^2^ (paired Wilcoxon test, *p* > 0.05; Figure S5a and Table S3). The microbial community on household surfaces was diverse. The top 10 most abundant bacterial genera were human-associated genera commonly linked to the gut (*Escherichia–Shigella*, *Faecalibacterium*, and *Lactobacillus*), oral cavity (*Staphylococcus* and *Streptococcus*), and skin (*Bacteroides* and *Corynebacterium*), as well as environmental genera (*Pseudomonas*, *Ralstonia*, and *Serratia*) (Figure S6a). Their relative abundances remained largely stable over time (paired Wilcoxon test, *p* > 0.05), although *Bacteroides*, *Faecalibacterium*, and *Streptococcus* abundances decreased significantly (paired Wilcoxon test, *p* < 0.01; Figure S7a).

All analyzed ions except ammonium were detected on surfaces in every household (Figure S6b and Table S4). Total ion concentrations varied significantly between days 5 and 30 (paired Friedman test, *p* < 0.05; Figure S5b), and the concentrations of all individual ions except fluoride also showed significant temporal changes (paired Friedman test, *p* < 0.05 for each; Figure S8a). Sodium was the most abundant ion throughout the study period (mole fraction range: 46.1 ± 19.9% on day 5 to 43.0 ± 16.3% on day 30; paired Wilcoxon test, *p* > 0.05), followed by chloride (mole fraction range: 18.0 ± 13.3% on day 5 to 23.9 ± 16.1% on day 30; paired Wilcoxon test, *p* > 0.05; Figure S6b). TOC accounted for a substantial proportion of the total carbon measured on household surfaces (day 5:73.0 ± 32.5%, day 30:78.1 ± 29.1%; paired Wilcoxon test, *p* > 0.05; Figure S6c). Surface TOC concentrations across all rooms varied significantly over time (paired Friedman test, *p* < 0.05; Figure S8b and Table S4).

### Individual Households and Oil-Based Cooking Frequency Influenced Surface Bacterial Communities

The influence of 16 factors, including three types of chemical constituents and 13 household- and occupant-related characteristics, on surface bacterial communities in each room across the 20 households was assessed. Wald tests were applied to MCMCglmm outputs to assess 16S rRNA gene copy numbers and α-diversity metrics, while bacterial community composition was evaluated using PERMANOVA based on Bray–Curtis dissimilarities. Among these factors, only individual households and oil-based cooking frequency significantly influenced multiple bacterial community parameters, including richness, the Shannon index, and community composition (*p* < 0.05) (Figure S9a). Individual households also significantly affected 16S rRNA gene copy numbers (*p* < 0.05) (Figure S9a). However, the four room types examined showed no significant influence on bacterial community parameters or abundances (*p* > 0.05) (Figure S9a).

Chemical constituents also played a key role in shaping the bacterial communities. TOC concentrations significantly influenced 16S rRNA gene copy numbers (*p* < 0.05) and bacterial composition (*p* < 0.01), while TIOC concentrations only affected bacterial composition (*p* < 0.05) (Figure S9a). Total ion concentrations significantly affected 16S rRNA gene copy numbers (*p* < 0.001) and richness (*p* < 0.05) (Figure S9a). TOC concentrations were significantly affected by room type, oil-based cooking frequency, window-opening frequency, cooling fan usage, exhaust fan usage, and cleaning frequency (*p* < 0.05) (Figure S9b). Total ion concentrations were primarily affected by individual households, cooling fan usage, exhaust fan usage, and cleaning frequency (*p* < 0.05), while TIOC concentrations were only significantly affected by individual households (*p* < 0.05) (Figure S9b).

### High Oil-Based Cooking Frequency Increased Surface TOC Concentrations

The influence of the oil-based cooking frequency on TOC concentrations on household surfaces was analyzed by comparing samples grouped into high, medium, and low oil-based cooking frequencies. High-cooking-frequency kitchens showed the highest surface TOC concentrations over time (Wald test, *p* = 0.009; Figure 1a). To further investigate this effect, a second sampling round was conducted in kitchens with well-controlled cooking frequencies using glass beads with and without coatings from laboratory-generated cooking fumes. TOC concentrations on uncoated beads were significantly higher in daily cooking kitchens than in noncooking kitchens (Wald test, *p* = 0.006; Figure 1b). Over 20 days, TOC concentrations on uncoated beads in daily cooking kitchens increased rapidly from 2.9 ± 1.3 μg/cm^2^ (day 5) to a peak of 7.6 ± 0.2 μg/cm^2^ (day 20) and then declined to 5.7 ± 2.3 μg/cm^2^ by day 30 (Figure 1b), suggesting degradation at higher loads. In noncooking kitchens, TOC concentrations on uncoated beads increased steadily from 2.0 ± 0.5 to 4.4 ± 0.3 μg/cm^2^ (days 5–25) and then stabilized. During recoating, TOC deposition on coated beads exceeded biotic and abiotic losses by 0.7 ± 0.5 μg/cm^2^ every 10 days (paired Wilcoxon test, *p* < 0.05). Coated beads in noncooking kitchens maintained intermediate TOC levels (4.2–5.5 μg/cm^2^) throughout, consistently falling between those of uncoated beads in both kitchen types (Figure 1b).

**Figure 1:**
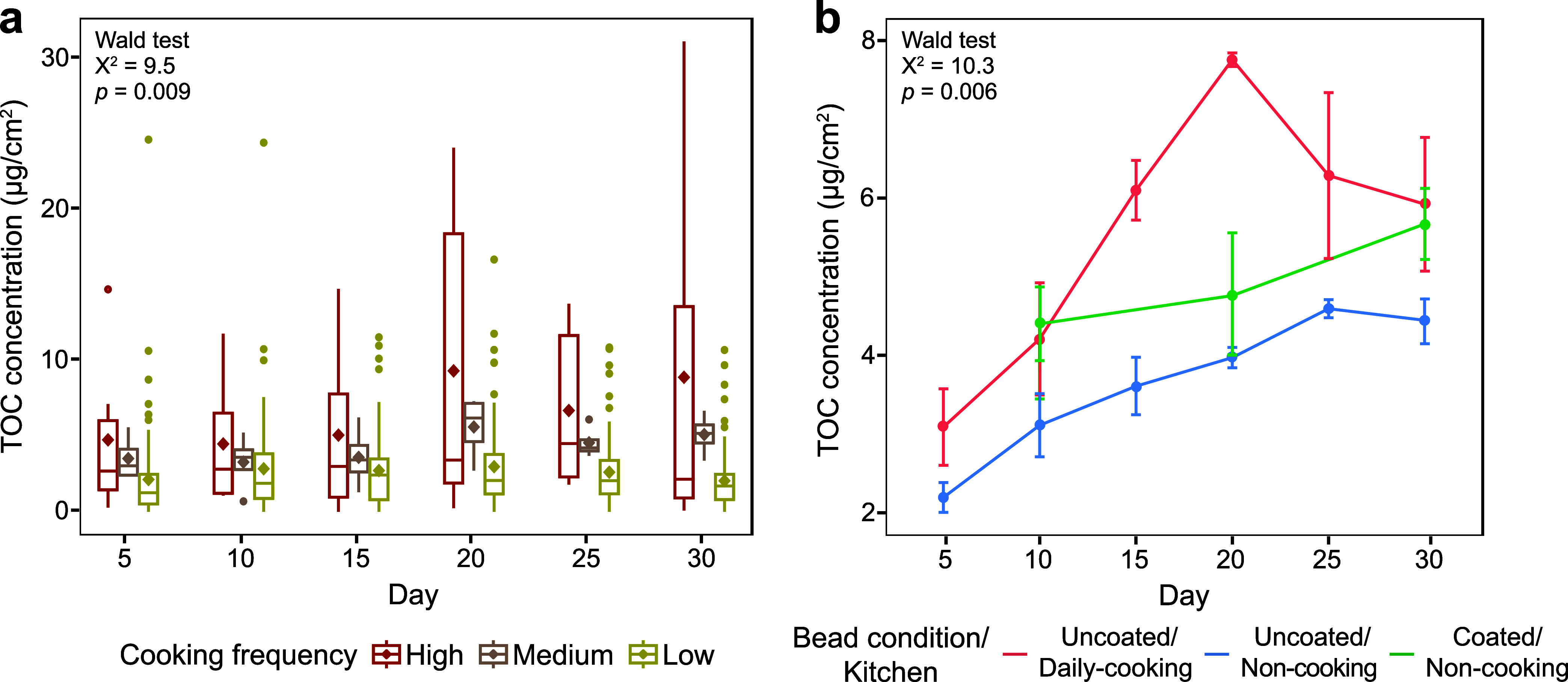
Temporal variations in total organic carbon (TOC) concentrations on household surfaces from the first and second sampling rounds. (a) Boxplot illustrating TOC concentrations across all sampled surfaces during the first sampling round. Each box represents the mean (diamond), median, first and third quartiles, and outliers (points beyond the whiskers), with the whiskers extending to 1.5 times the interquartile range. (b) Line plot showing TOC concentrations on kitchen surfaces over time during the second sampling round, with error bars representing the standard error. Statistical differences over time among the three groups (defined by cooking frequency in the first round and by cooking frequency with different bead conditions in the second round) were assessed using the Wald test.

To identify the organic compounds on kitchen surfaces, beads from the second sampling round were analyzed using GC-MS. Overall, 106 compounds were detected across all samples, primarily comprising alkanes, fatty acids, and PAHs. Similar numbers of compounds were identified across sample types: 99 in daily cooking kitchens and 97 and 98 in noncooking kitchens on uncoated and coated beads, respectively. However, the composition of these compounds varied significantly by the oil-based cooking frequency and bead condition (PERMANOVA, *p* = 0.001) (Figure S10 and Table S5), with certain compounds being exclusive to specific conditions. For example, glycidyl heptadecenoate, dotriacontane, furan, and carbonic acid were found exclusively in daily cooking kitchens. Tridecane was only detected on coated beads in noncooking kitchens, while dodecanol was found exclusively on uncoated beads in noncooking kitchens (Table S5). Some compounds, such as octadecenoic acid and octadecane, were only found on uncoated beads in daily cooking kitchens and on coated beads in noncooking kitchens. Compounds such as tetracosane were exclusively found in noncooking kitchens, irrespective of bead condition (Table S5).

### High Oil-Based Cooking Frequency Increased Surface Bacterial Abundance

The influence of the oil-based cooking frequency on bacterial abundance was analyzed across the two sampling rounds. In the first round, no significant differences were observed in 16S rRNA gene copy numbers over time among the three groups categorized by oil-based cooking frequency (Wald test, *p* > 0.05; Figure 2a). However, in the second round, 16S rRNA gene copy numbers increased significantly over time on uncoated beads in daily cooking kitchens relative to coated and uncoated beads in noncooking kitchens (Wald test, *p* = 0.002; Figure 2b). In daily cooking kitchens, gene copy numbers increased steadily, peaking at 3.9 ± 0.1 log_10_ copies/cm^2^ by day 25, compared with 2.5 ± 0.5 log_10_ copies/cm^2^ in noncooking kitchens (Wald test, *p* = 0.002; Figure 2b). Coated beads in noncooking kitchens also exhibited a gradual increase in 16S rRNA gene copy numbers, with levels reaching between those on uncoated beads from cooking and noncooking kitchens (Figure 2b).

**Figure 2:**
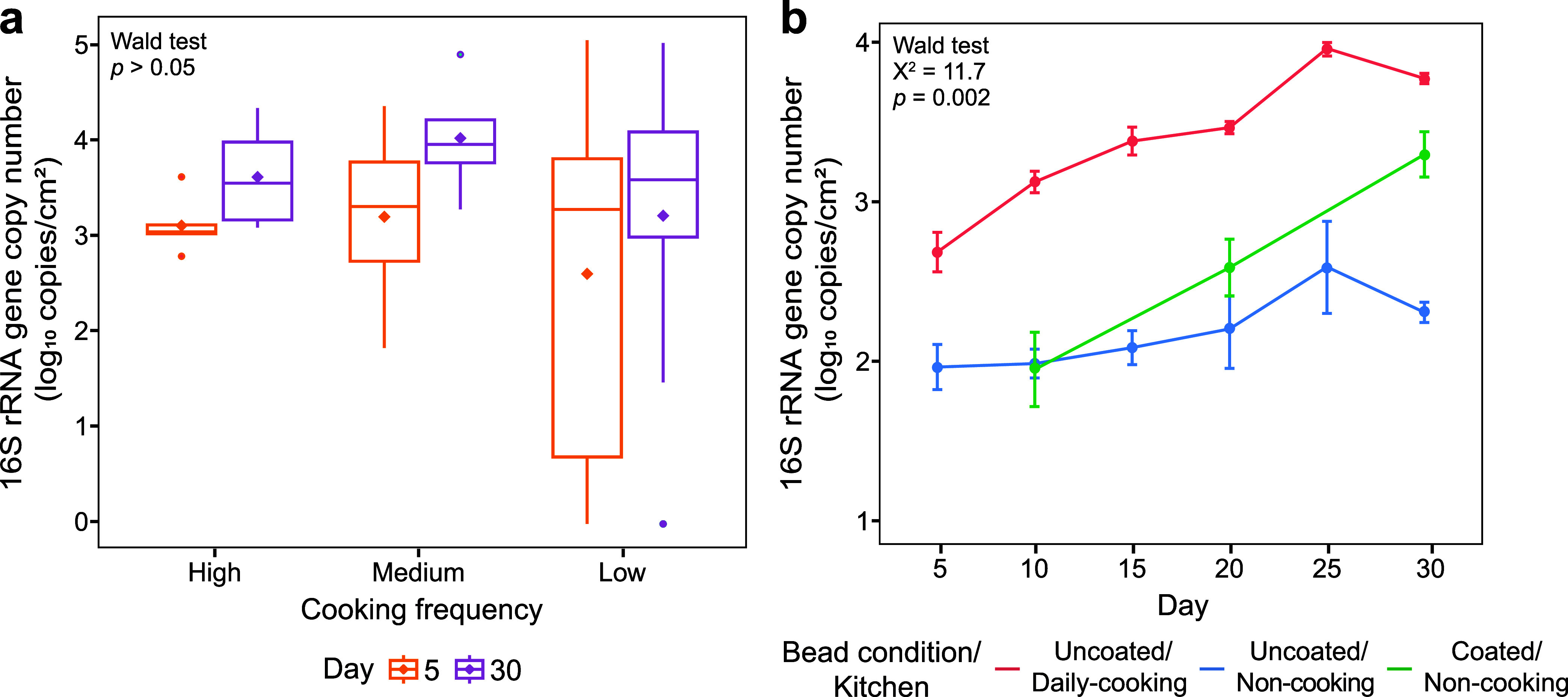
Bacterial abundance on household surfaces from the first and second sampling rounds. (a) Boxplot illustrating 16S rRNA gene copy numbers across all sampled surfaces during the first sampling round. Each box represents the mean (diamond), median, first and third quartiles, and outliers (points beyond the whiskers), with the whiskers extending to 1.5 times the interquartile range. (b) Line plot showing changes in the 16S rRNA gene copy numbers on kitchen surfaces over time during the second sampling round, with error bars indicating the standard error. Statistical differences over time among the three groups (defined by cooking frequency in the first round and by cooking frequency with different bead conditions in the second round) were assessed using the Wald test.

### High Oil-Based Cooking Frequency Altered Surface Bacterial Community Diversity and Composition

The influence of the oil-based cooking frequency on bacterial diversity and composition was further assessed in the two sampling rounds. In the first round, significant differences in α-diversity metrics, specifically richness (Wald test, *p* = 0.04) and the Shannon index (Wald test, *p* = 0.03), were observed between days 5 and 30 in high-cooking-frequency kitchens (Figure S11a,b). In the second round, daily cooking kitchens showed a significantly higher richness (Wald test, *p* = 0.01; Figure 3a) and Shannon index over time than noncooking kitchens (Wald test, *p* < 0.001; Figure 3b). Peaks in richness and Shannon index occurred at different times across the three groups and under different bead conditions. In daily cooking kitchens, bacterial diversity peaked on day 15, whereas in noncooking kitchens, peaks occurred later for both uncoated and coated beads (Figure 3a,b).

**Figure 3:**
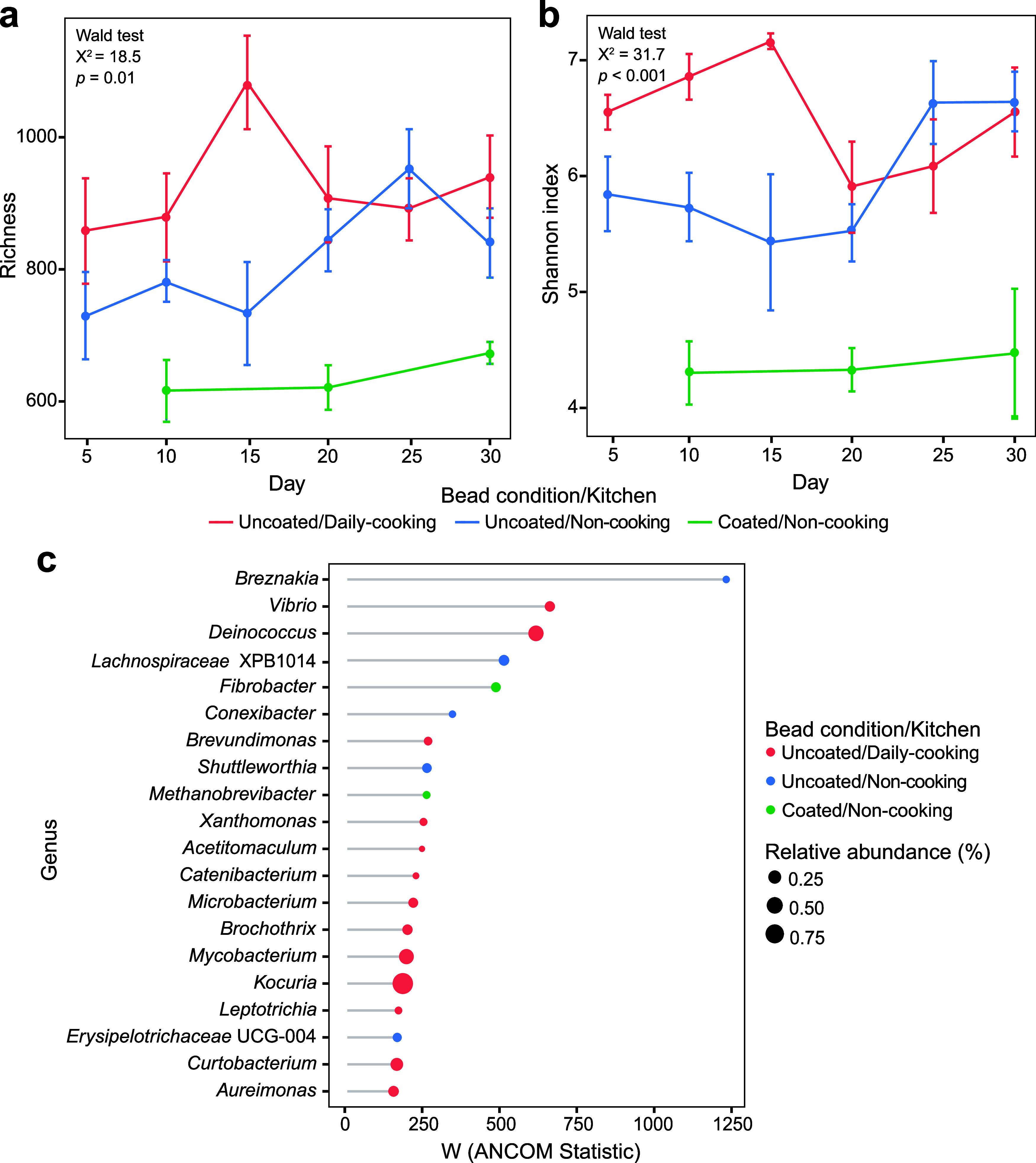
Bacterial diversity and composition on kitchen surfaces from the second sampling round. (a, b) Line plots illustrating changes in α diversity over time, measured using (a) richness and (b) the Shannon index, with error bars representing the standard error. Statistical differences over time among the three groups of kitchens with varying cooking frequencies and bead conditions were assessed using the Wald test. (c) Top 20 differentially abundant genera identified across the three groups with varying cooking frequencies and bead conditions based on an ANCOM-BC. Circle size represents the average relative abundance of each genus within its respective group. The *x*-axis (W statistic) indicates the magnitude of differential abundance, with larger absolute values representing stronger differences between groups.

The bacterial community composition varied significantly with the oil-based cooking frequency, as shown in the first sampling round (PERMANOVA, *R* ^2^ = 0.03, *p* = 0.003) and further supported by the second round (PERMANOVA, *R* ^2^ = 0.20, *p* = 0.001). During the first round, differential analysis on days 5 and 30 revealed significant variations in the relative abundances of bacterial genera among the three cooking-frequency groups. On day 5, of the top 20 differentially abundant genera, only eight were associated with high-cooking-frequency kitchens (Figure S11c). However, by day 30, 18 of these 20 genera were associated with high-cooking-frequency kitchens (Figure S11d). In the second round, by day 30, 13 of the 20 genera (*e.g.*, *Vibrio*, *Deinococcus*, and *Brevundimonas*) were found on uncoated beads in daily cooking kitchens. The remaining seven genera were associated with noncooking kitchens: five (*e.g.*, *Breznakia*, *Conexibacter*, and *Shuttleworthia*) found exclusively on uncoated beads and two (*Fibrobacter* and *Methanobrevibacter*) on coated beads (Figure 3c).

To further investigate differences in bacterial community structure and interactions on kitchen surfaces, a co-occurrence network analysis was conducted comparing kitchens with low and high oil-based cooking frequencies on days 5 and 30 using taxa from the first sampling round (Table S6). Compared with low-cooking-frequency kitchens, high-cooking-frequency kitchens exhibited a significant increase in network density and integration, with bacterial taxa displaying over 10 times more nodes and hundreds of times more edges on days 5 and 30. Additionally, the average degree of connectivity was approximately 20 times higher than that in low-frequency cooking kitchens, indicating a more interconnected microbial community and increased bacterial interactions. Notably, all correlations between bacterial taxa in low-cooking-frequency kitchens were positive, regardless of sampling day, while only ∼50% were positive in high-cooking-frequency kitchens, suggesting a shift toward more competitive microbial interactions.

### Strong Interactions between Cooking-Derived Organic Compounds and Bacterial Taxa

A network analysis was performed using the organic compounds identified in the second sampling round to examine their interactions with bacterial communities in the three groups of kitchen surfaces categorized by cooking frequency and bead condition (Figure 4 and Table S7). The results revealed distinct interaction patterns and connectivity among the sample types. Uncoated beads from daily cooking kitchens exhibited fewer nodes and edges but a higher average weighted node degree than uncoated beads from noncooking kitchens, suggesting stronger interactions between dominant organic compounds and bacterial taxa. However, coated beads from noncooking kitchens showed the highest numbers of nodes, edges, and modularity, exceeding uncoated beads from both daily cooking and noncooking kitchens. Interaction network composition also varied by condition: 67.1% of correlations between organic compounds and genera were positive in daily cooking kitchens with uncoated beads, 62.2% in noncooking kitchens with coated beads, and 50% in noncooking kitchens with uncoated beads.

**Figure 4:**
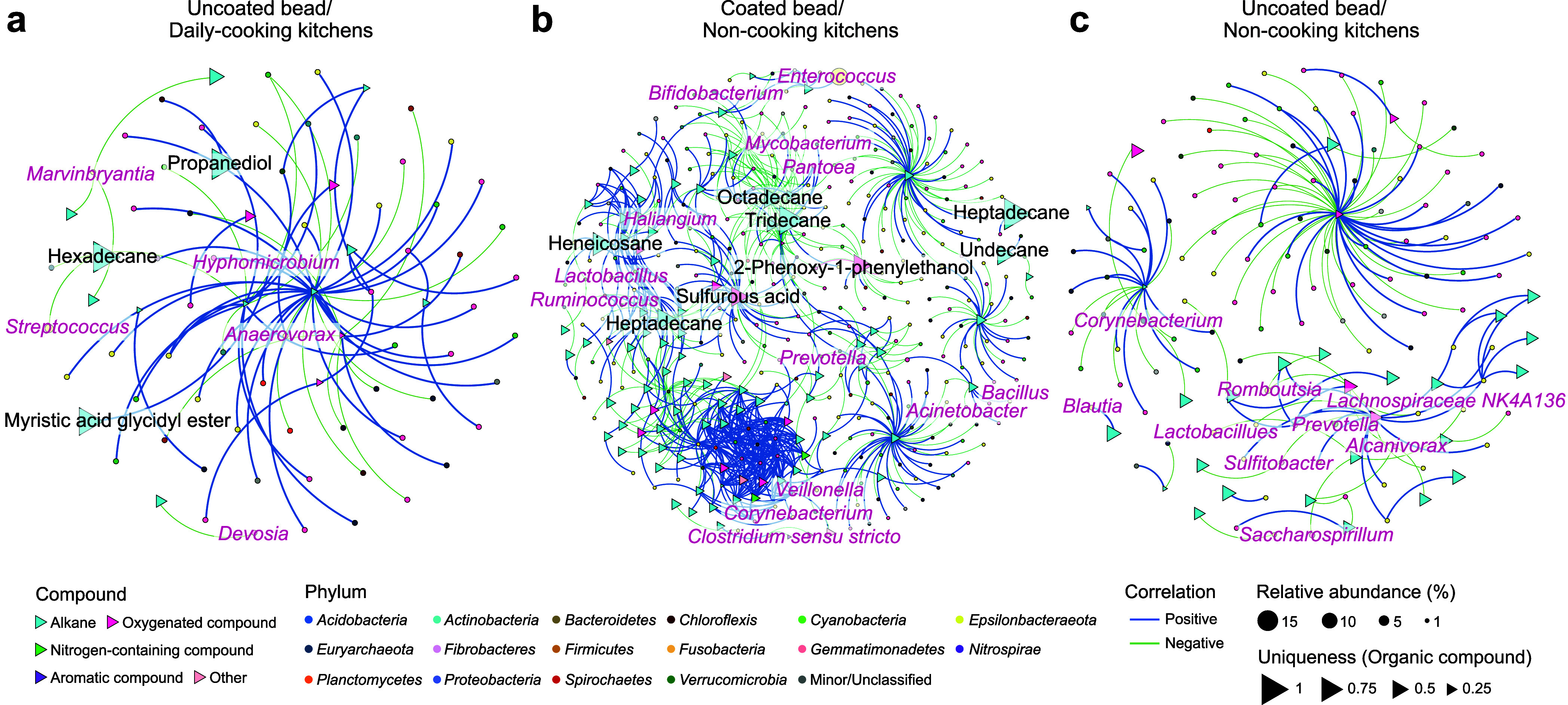
Networks of amplicon sequence variants (ASVs) and organic compounds on kitchen surfaces from the second sampling round. Nodes represent ASVs (circles) and organic compounds (triangles), with sizes indicating ASV relative abundance or the uniqueness of an organic compound (its relative abundance at a specific cooking frequency relative to its total abundance across all cooking frequencies and bead conditions). Node colors correspond to the top 20 most abundant phyla across the three groups of kitchens with varying cooking frequencies and bead conditions. Edges represent significant correlations (Spearman’s |ρ| > 0.8), with edge thickness proportional to the ρ values and colors denoting positive or negative correlations. Labels indicate ASVs classified at the genus level with relative abundances >0.15% and the identified organic compounds with uniqueness >0.05.

To further explore the taxa strongly correlated with organic compounds, their representation in the networks from kitchens with varying cooking frequencies and bead conditions was analyzed (Figure S12a,b). *Proteobacteria* and *Firmicutes* were the most represented phyla, accounting for 31 ± 4.9% and 26.3 ± 4.8% of network nodes, respectively, across all samples, regardless of the cooking frequency or presence of cooking fumes. Within *Proteobacteria*, *Alphaproteobacteria* showed the strongest correlations, with 11.7 ± 6.2% positive and 16.9 ± 2.9% negative associations. In *Firmicutes*, *Clostridia* had the highest positive (17.8 ± 7.6%) and negative (21.3 ± 3.4%) correlations with organic compounds. Among the detected chemicals, alkanes exhibited the strongest correlations with ASVs, with 85 ± 11.4% positive and 74 ± 5.6% negative, regardless of cooking frequency or exposure to cooking fumes (Figure S12c). In daily cooking kitchens, three organic compounds, namely, propanediol, heneicosane, and myristic acid glycidyl ester, had high uniqueness scores (>0.5) and significant ASV correlations. For example, myristic acid glycidyl ester was positively correlated with *Hyphomicrobium*, while heneicosane was negatively correlated with *Streptococcus.* In noncooking kitchens, eight compounds on coated beads also had high uniqueness scores and significant ASV correlations. Notably, tridecane was positively correlated with *Mycobacterium*, and heneicosane was negatively correlated with *Haliangium*. In contrast, compounds on uncoated beads in noncooking kitchens showed significant ASV correlations only when uniqueness scores were low (<0.5), suggesting they were common across conditions and lacked specificity.

### Alkanes as Key Drivers Shaping Bacterial Composition

A db-RDA was conducted to identify the organic compounds most strongly linked to differences in surface bacterial composition across the three kitchen groups categorized by cooking frequency and bead conditions during the second sampling round. Of the GC-MS-detected 106 compounds, seven alkanes were the most influential on uncoated beads in daily cooking kitchens and both coated and uncoated beads in noncooking kitchens (ANOVA, *p* < 0.01), explaining 29.9% of the variance in bacterial communities along the first two RDA axes (Figure 5). In daily cooking kitchens, octane (ANOVA, *p* = 0.005) was the most significant driver of the bacterial community composition. In noncooking kitchens, undecane (ANOVA, *p* = 0.007) and tridecane (ANOVA, *p* = 0.008) were key contributors to community differences on coated beads, while several alkanes, including heptadecane (ANOVA, *p* = 0.002), docosane (ANOVA, *p* = 0.003), tetracosane (ANOVA, *p* = 0.001), and tricontane (ANOVA, *p* = 0.001), significantly influenced bacterial composition on uncoated beads.

**Figure 5:**
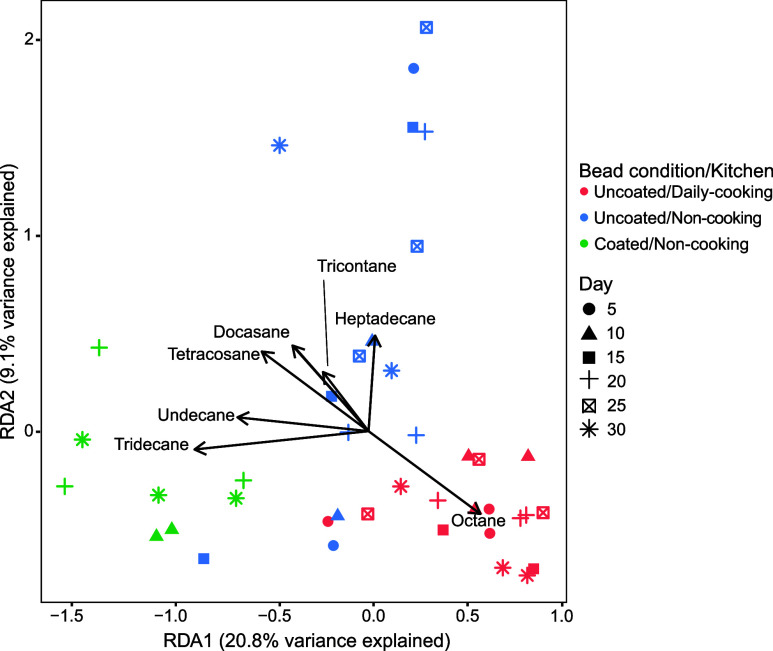
Distance-based redundancy analysis of the bacterial community structure influenced by organic compounds from the second sampling round. Vectors represent significant organic compounds (*p* < 0.01, ANOVA) associated with differences in bacterial composition across the three groups of kitchens with varying cooking frequencies and bead conditions. Vector length and direction indicate the strength and correlation of each compound with the microbial community structure.

## Discussion

The effects of surface chemical and biological constituents in indoor settings on occupant health have recently received increasing attention.^6,10^ However, most field studies have examined these factors in isolation, focusing on surface-associated chemicals or bacterial communities at specific time points,^21–24^ leaving their interactions largely underexplored. To explore these interactions, we conducted two rounds of field investigations in households using uncoated and cooking-fume-coated glass beads. Our findings revealed that frequent cooking significantly increased bacterial abundance, reduced bacterial diversity, and altered the bacterial composition on indoor surfaces *via* the release and surface deposition of organic compounds.

We identified human-associated bacteria on household surfaces, supporting prior findings that up to 88% of surface bacteria originate from humans.^30–32^ Each individual sheds approximately 10^7^ bacteria per hour from clothing, breath, and skin, significantly contributing to surface bacterial loads.^8,33,34^ We also detected environmental genera presumably introduced through infiltration, natural ventilation,^35,36^ or passive transport *via* clothing or skin.^37,38^ Sodium and chloride, the most abundant ions on household surfaces, likely originate from human sweat,^39^ cooking activities,^40^ and proximity to seawater,^41^ consistent with prior research.^2^ Airborne ammonia concentrations in households average at 0.21 mg/m^3^,^42^ primarily contributed by cleaning products or human skin.^43^ However, no ammonium was detected on surfaces, possibly due to volatilization.^2^ We observed significantly higher concentrations of organic compounds than inorganic compounds on household surfaces, reflecting the dominant influence of indoor activities.^44^

The organic compounds, including alkanes, fatty acids, and PAHs, accumulating on surfaces over time in high-cooking-frequency kitchens may have predominantly originated from cooking oils.^7,45^ In noncooking kitchens, these compounds probably originated from human skin shedding,^46^ cleaning product residues,^47^ or other indoor and outdoor contaminants,^48^ although their contribution to the total organic load was low. In the households studied, TOC concentrations in high-cooking-frequency kitchens were nearly double those in noncooking kitchens after 25 days, indicating that a substantial portion of the accumulated TOC was attributable to cooking. Within just a few hours of cooking, TOC concentrations on surfaces could be 3-fold those in noncooking periods,^7^ highlighting cooking as a significant and rapid contributor to TOC. Among cooking-derived compounds, alkanes could particularly promote bacterial growth due to their high carbon content and simple structure.^12^ The increase in bacterial abundance closely aligned with the increase in TOC concentrations, indicating that cooking-derived organic compounds directly contributed to the TOC concentrations, supporting bacterial proliferation. Kitchen surfaces, known to harbor over 300 times more bacteria than other household surfaces,^49^ showed an average of 1.4-fold increase in bacterial abundance in active cooking kitchens from day 5 to 30. In high-frequency cooking environments, more abundant bacterial communities likely accelerated organic matter degradation, outpacing deposition and contributing to the decline in TOC concentrations after the day 20 peak. Hence, the potential for bacterial proliferation on kitchen surfaces should not be underestimated. In noncooking kitchens, uncoated glass beads with low organic content did not support significant bacterial growth, while cooking-fume-coated beads showed lower bacterial abundance than those in high-cooking-frequency kitchens. This suggests that only some indigenous bacteria in noncooking environments are adapted to utilize cooking-derived organic compounds.^23^

Frequent cooking causes organic compound accumulation on kitchen surfaces, increasing bacterial abundance and reducing microbial diversity over time. During the first 15 days, bacterial diversity initially increased as compounds such as alkanes provided carbon and energy sources to support diverse bacterial growth. Subsequently, diversity declined, probably due to metabolic stress caused by the accumulation of oxidation products of the original organic compounds. For example, the oxidation of unsaturated fatty acids generates reactive oxygen species (*e.g.*, peroxidic products),^50^ which can damage lipids, proteins, and DNA, eventually causing lethal damage to some bacteria.^51^ However, certain taxa, *e.g.*, *Vibrio* species, were differentially abundant under these conditions, probably due to their ability to produce oxidative-stress-countering enzymes like superoxide dismutase and catalase.^52^ Similarly, *Deinococcus* species likely persisted by relying on robust DNA repair mechanisms,^53^ which may have enabled their survival in environments rich in cooking-derived organic compounds and their oxidation products. Thus, the overall bacterial abundance increased even as diversity declined, reflecting nutrient-driven selection observed in other built environment studies, where newly available resources favor specific microbial subsets.^54^ Conversely, coated beads in noncooking kitchens showed lower and relatively stable diversity over time, presumably due to limited initial diversity and the absence of taxa capable of countering oxidative stress from cooking-derived organic compounds.^55^ On uncoated beads in noncooking kitchens, bacterial diversity gradually increased, suggesting that organic compounds from noncooking sources lacked the same toxicity level as those from cooking sources.^56^

The dominance of cooking-derived organic compounds in high-cooking-frequency kitchens promotes extensive interactions with bacterial taxa, as reflected by the higher average weighted node degree in their networks compared to uncoated beads in noncooking kitchens. Genera such as *Mycobacterium*, known for high dehydrogenase activity, were strongly associated with specific alkanes (*e.g.*, dimethyldodecane),^57^ suggesting potential utilization of these compounds as energy sources. In contrast, some alkanes (*e.g.*, heneicosane) have reported antimicrobial properties, though their mechanisms remain unclear and warrant further investigation.^58^ Enhanced bacterial interactions in high-cooking-frequency kitchens may result from the degradation of organic compounds, which facilitates resource exchange among diverse bacterial taxa.^59^ However, the increase in negative interactions may reflect intensified competition as bacteria increasingly rely on shared nutrients over time.^54^ In noncooking kitchens, coated beads formed a network with a relatively high number of nodes and edges linking organic compounds to bacterial taxa. However, the proportion of positive interactions was similar to that of uncoated beads in daily cooking kitchens, suggesting that cooking-derived organic compounds, regardless of their source, can support microbial communities, with both kitchen types showing comparable metabolic utilization. In contrast, the network on uncoated beads in noncooking kitchens showed fewer positive interactions, indicating that noncooking-related organic compounds may be less supportive of bacterial growth.

This study highlights the influence of cooking-derived organic compounds on surface bacterial communities but has several limitations. First, although several households were analyzed, logistical constraints limited the inclusion of additional households with more varied interior designs, cooking practices, and geographic locations. Incorporating these variables may help to determine whether similar changes occur across different settings. Second, in the recoating experiments in noncooking households, beads were recoated with fresh cooking fumes every 10 days, unlike the daily exposure experienced in active cooking kitchens. This longer interval may have reduced the selection pressure on microbial communities relative to that in daily cooking environments. Nevertheless, the results demonstrated that even reduced exposure significantly altered the microbial communities on coated beads compared with those on uncoated beads, highlighting the strong impact of cooking-derived organic compounds in shaping microbial composition even in noncooking settings. Third, while 16S rRNA gene sequencing provided valuable taxonomic insights into how cooking frequency influences microbial diversity and composition, future studies may benefit from incorporating metagenomic sequencing, enabling a deeper analysis of microbial metabolic functions and genome reconstruction, including the identification of potential pathogens.^60^ Lastly, the analysis of organic compounds may have excluded certain key molecules, and the interactions between microbial taxa and surface chemicals are presumably more complex than those depicted. The use of GC-MS also limits detection primarily to semivolatile organic compounds, representing only a fraction of indoor organic compounds. Future research employing high-throughput chemical analysis^61^ and other analytical techniques (*e.g.*, liquid chromatography-high-resolution mass spectrometry)^62,63^ may help identify a diverse range of compounds across various volatility levels, providing a deeper understanding of the microbiology–surface chemistry interplay.

## Environmental Implications

This study highlights how frequent oil-based cooking promotes bacterial growth and alters the microbial diversity and composition on kitchen surfaces. While the use of a 16S rRNA gene fragment limited the identification of specific pathogens, their presence cannot be ruled out, as they may thrive in the presence of cooking-derived compounds. Kitchens are consistently identified as pathogen reservoirs,^64^ with quantitative risk assessments showing that pathogens such as , , and often exceed health risk standards on high-touch surfaces like refrigerator handles, cupboard handles, and sinks.^65^ These findings underscore the need to reduce the accumulation of cooking-derived compounds to curb bacterial proliferation. Practices such as using exhaust fans during cooking can decrease occupants’ exposure to harmful fumes^66^ and minimize organic compound deposition onto surfaces.^67^ Besides cleaning countertops after handling raw meat to eliminate foodborne pathogens,^68^ this study highlights the importance of regular cleaning to control bacterial growth stemming from cooking fumes, with the ultimate goal being to ensure a healthy environment for occupants. This study demonstrated how routine activities, such as cooking, can significantly shape indoor microbial communities. Similar chemical–biological interactions potentially result from other household activities and warrant further investigation. Further research should also explore how microbial changes driven by cooking and other indoor activities affect the occupant microbiomes, including the gut,^69^ skin,^70^ and respiratory tract microbiomes.^71^

## Supplementary Material





## Notes

Schematic overview of the two sampling rounds; geographical locations of the households; concentrations of constituents in control samples from the recoating procedures; TOC concentrations in samples for abiotic factor controls; concentrations of constituents on surfaces from the first sampling round; compositions of constituents on surfaces from the first sampling round; comparison of constituent concentrations on surfaces from the first sampling round; effects of various factors on bacterial communities and chemical concentrations on surfaces during the first sampling round; PCoA plot of organic compound compositions on surfaces from the second sampling round; bacterial diversity and composition on surfaces from the first sampling round; phylum and organic compound representation in the networks of ASVs and organic compounds on surfaces from the second sampling round; assessment of PCR inhibition; detailed analysis methods (PDF)
Household characteristics from the first sampling round; 16S rRNA gene copy numbers in surface samples from the first and second sampling rounds; concentrations of chemical constituents in surface samples from the first and second sampling rounds; average relative abundance and uniqueness of organic compounds from the second sampling round; network parameters of ASVs on surfaces from the first sampling round; and network parameters of ASVs and organic compounds on surfaces from the second sampling round (XLSX)
