# Supplementary material for: Cooking-Derived Organic Compounds Shape Bacterial Communities on Household Surfaces
Source: Environ Sci Technol. 2025 Jul 7;59(27):13924–34. doi: 10.1021/acs.est.5c02724 (PMC12269069; doi:10.1021/acs.est.5c02724)
Supplement: Supplementary file 2 [file es5c02724_si_002.pdf]

Supplementary Information

Cooking-derived organic compounds shape bacterial communities on household surfaces

Wing Lam Chan[1], Shicong Du[1], Huiju Lin[2], Yin Hau Lam[1], Jason Chun-Ho Lam[3,4], Theodora Nah[3,4], and Patrick K. H. Lee[3,4]*

[1]School of Energy and Environment, City University of Hong Kong, Hong Kong SAR, China

[2]State Key Laboratory of Marine Environmental Health, City University of Hong Kong, Hong Kong SAR, China

[3]School of Energy and Environment and State Key Laboratory of Marine Environmental Health, City University of Hong Kong, Hong Kong SAR, China

[4]Low-Carbon and Climate Impact Research Centre, City University of Hong Kong, Hong Kong SAR, China

Correspondence: *B5423, Yeung Kin Man Academic Building, School of Energy and Environment, City University of Hong Kong, Tat Chee Avenue, Kowloon, Hong Kong SAR, China; E-mail: patrick.kh.lee@cityu.edu.hk; Tel: (852) 3442-4625; Fax: (852) 3442-0688.

Supplementary Information includes 24 pages, 14 figures, 3 text sections, and 6 tables.

Table of Contents

Figure S1. Schematic overview of the two sampling rounds. 3

Figure S2. Geographical locations of the sampled households in Hong Kong. 4

Figure S3. Concentrations of chemical and biological constituents in control samples assessing the recoating procedures. 5

Figure S4. Total organic carbon (TOC) concentrations in control samples assessing abiotic influences. 6

Figure S5. Concentrations of biological and chemical constituents on household surfaces from the first sampling round. 7

Figure S6. Compositions of biological and chemical constituents on household surfaces on days 5 and 30 from the first sampling round. 8

Figure S7. Comparison of the top 10 most abundant genera on household surfaces between days 5 and 30 of the first sampling round, with relative abundances illustrated using boxplots across all sampled household surfaces. 9

Figure S8. Temporal trends in surface chemical constituents from the first sampling round. 10

Figure S9. Influences of various factors on bacterial communities and chemical concentrations on household surfaces during the first sampling round. 11

Figure S10. Principal coordinates analysis (PCoA) plot of organic compound compositions on kitchen surfaces from the second sampling round. 12

Figure S11. Bacterial diversity and composition on household surfaces from the first sampling round. 13

Figure S12. Representation of phyla and organic compounds in the networks of amplicon sequence variants (ASVs) and compounds on kitchen surfaces from the second sampling round. 14

Figure S13. Assessment of PCR inhibition using serial dilutions. 15

Figure S14. Rarefaction analysis of amplicon sequence variant (ASV) counts. 16

Text S1. Detailed procedures for chemical analysis 17

Text S2. Detailed procedures for genomic DNA extraction, quantitative polymerase chain reaction (qPCR), and amplicon sequencing 18

Text S3. Detailed procedures for bioinformatics and statistical analyses 20

References 23

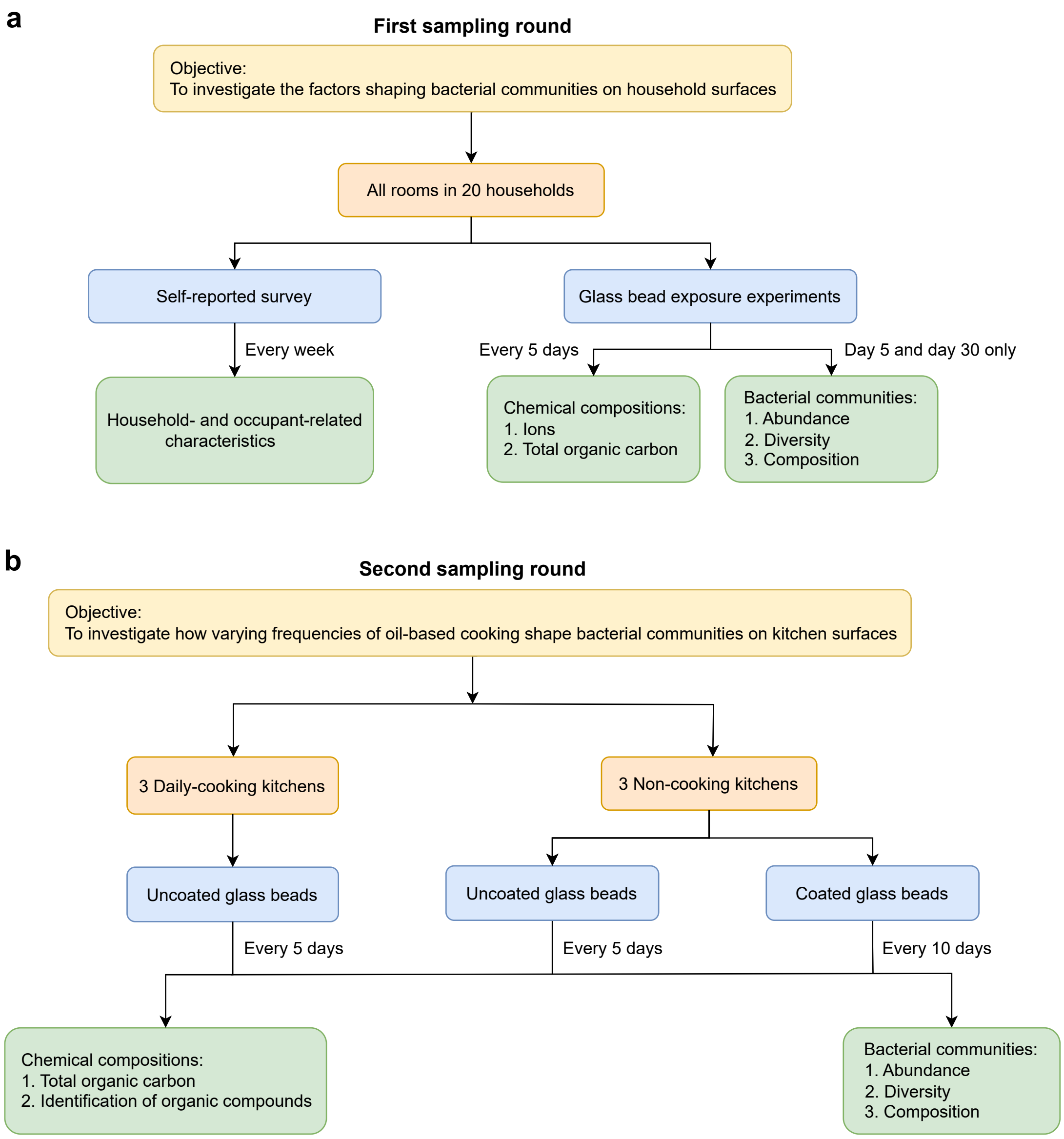

Figure S1. Schematic overview of the two sampling rounds. (a) The first round used an exploratory design to identify key drivers of bacterial communities on household surfaces, revealing oil-based cooking as the primary factor. **(b)** The second round applied controlled experimental conditions with different cooking frequencies and bead conditions, and increased temporal resolution, to more precisely assess the impact of oil-based cooking on kitchen surface bacterial communities.

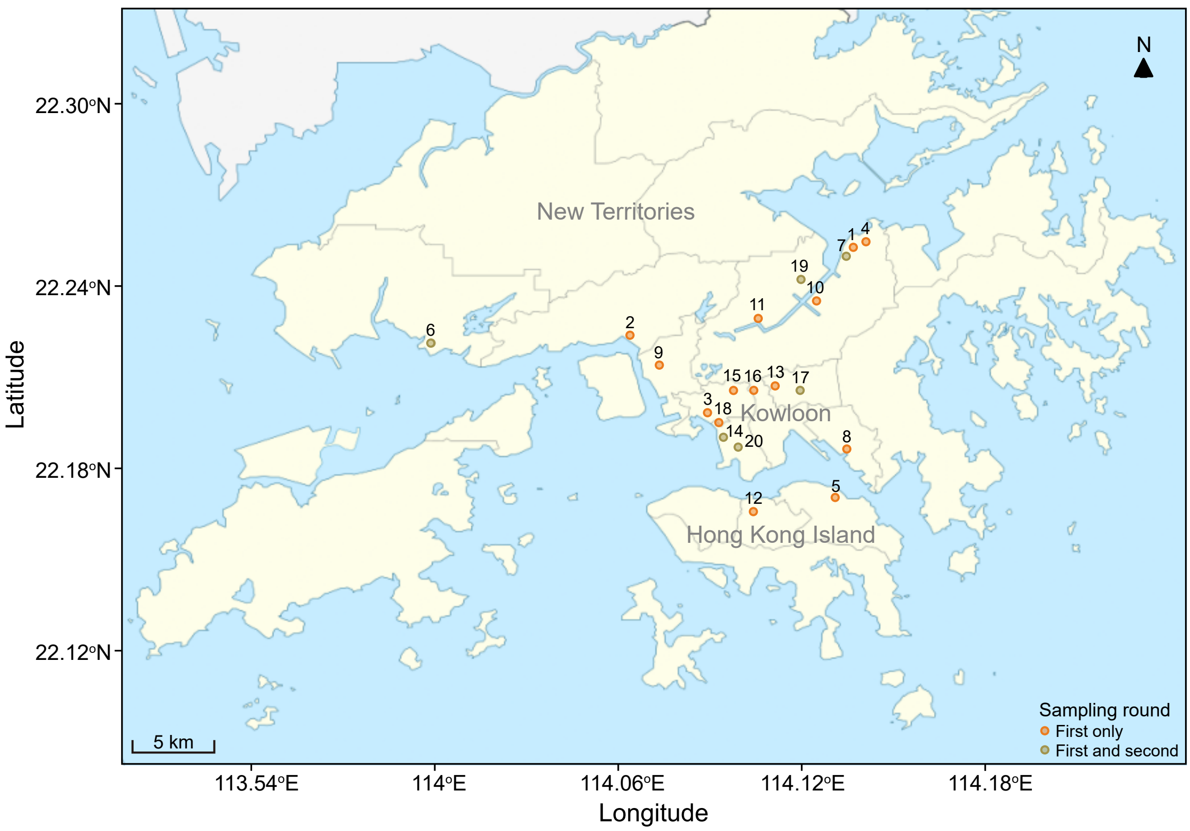

Figure S2. Geographical locations of the sampled households in Hong Kong. Surface samples were collected from all rooms in 20 households during the first sampling round. The second round focused on kitchen surfaces in six households selected based on cooking activities observed in the first round.

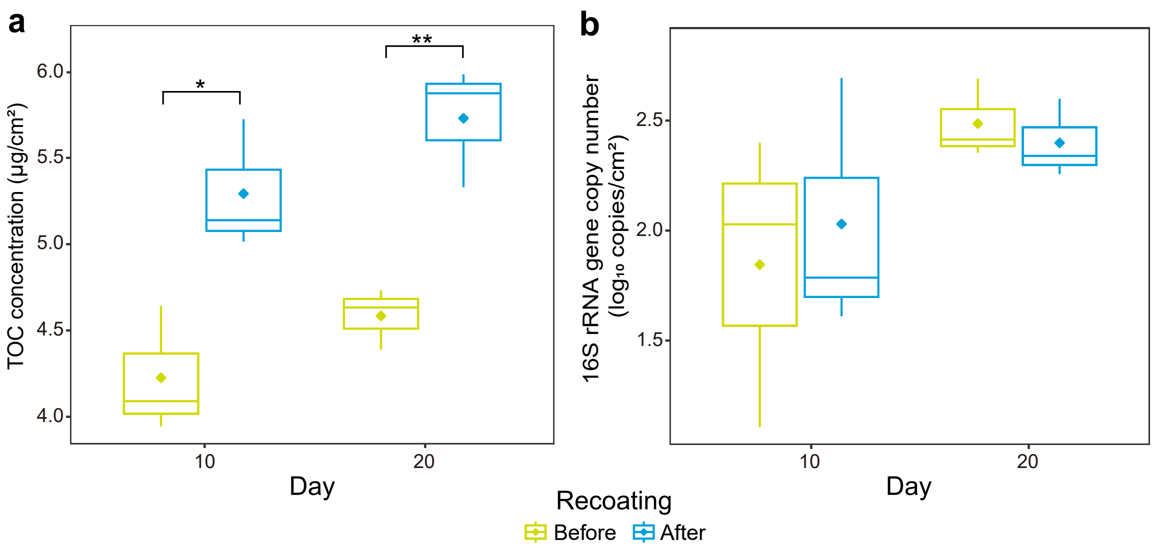

Figure S3. Concentrations of chemical and biological constituents in control samples assessing the recoating procedures. (a, b) Boxplots illustrating **(a)** TOC concentrations and **(b)** 16S rRNA gene copy numbers on bead surfaces before and after recoating with cooking fumes on days 10 and 20 of the second sampling round. Each box represents the mean (diamond), median, and first and third quartiles, with the whiskers extending to 1.5 times the interquartile range. Statistical differences were evaluated using the paired Wilcoxon test (**$p < 0.01$; *$p < 0.05$). No significant changes were observed in 16S rRNA gene copy numbers at either time point.

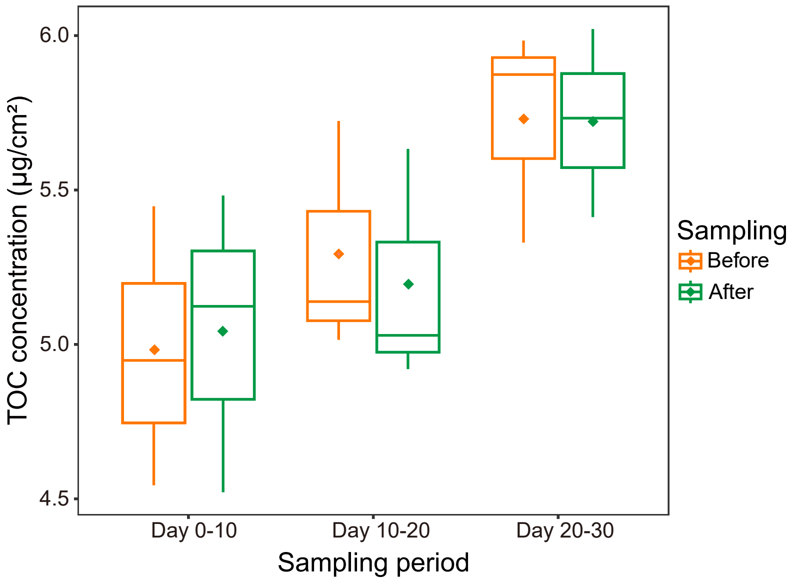

Figure S4. Total organic carbon (TOC) concentrations in control samples assessing abiotic influences. Boxplots showing TOC concentrations on coated or recoated beads before and after 10 days of sampling in closed-lid boxes. Each box represents the mean (diamond), median, and first and third quartiles, with the whiskers extending to 1.5 times the interquartile range. No significant changes in TOC concentrations were observed before and after sampling for each 10-day period (paired Wilcoxon test, $p > 0.05$).

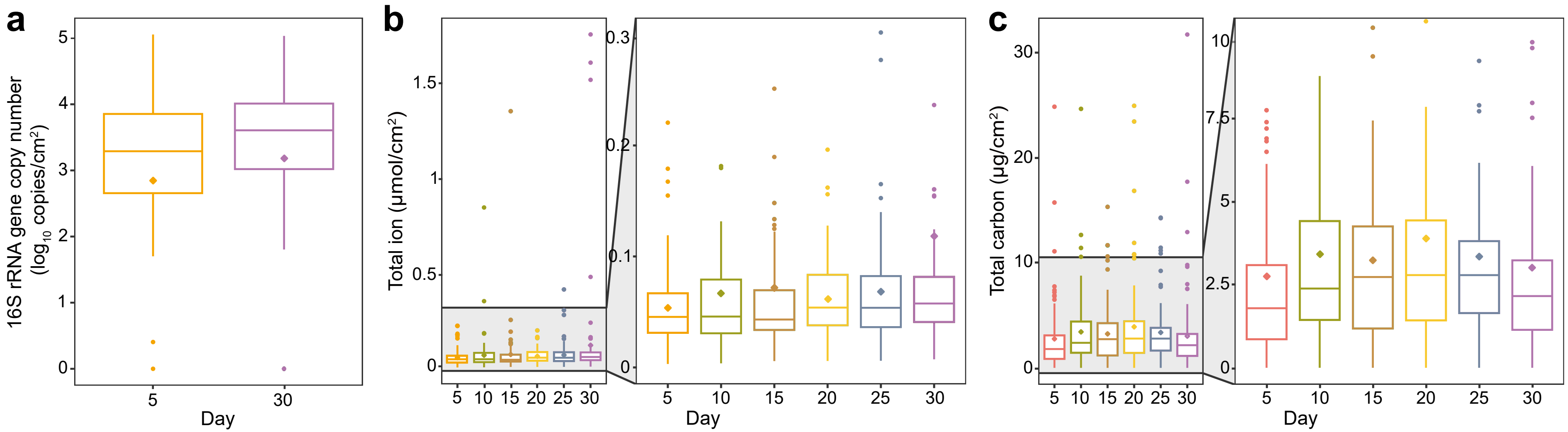

Figure S5. Concentrations of biological and chemical constituents on household surfaces from the first sampling round. (a, b) Boxplots illustrating **(a)** 16S rRNA gene copy numbers, **(b)** total ion concentrations, and **(c)** TOC concentrations. A zoomed-in panel highlights data in the lower range. Each box represents the mean (diamond), median, first and third quartiles, and outliers (points beyond the whiskers), with the whiskers extending to 1.5 times the interquartile range. No significant changes were detected in any biological or chemical constituents across all collection time points, as assessed using paired Wilcoxon tests for 16S rRNA gene copy numbers ($p > 0.05$) and paired Friedman test for total ion and total carbon (both $p < 0.05$).

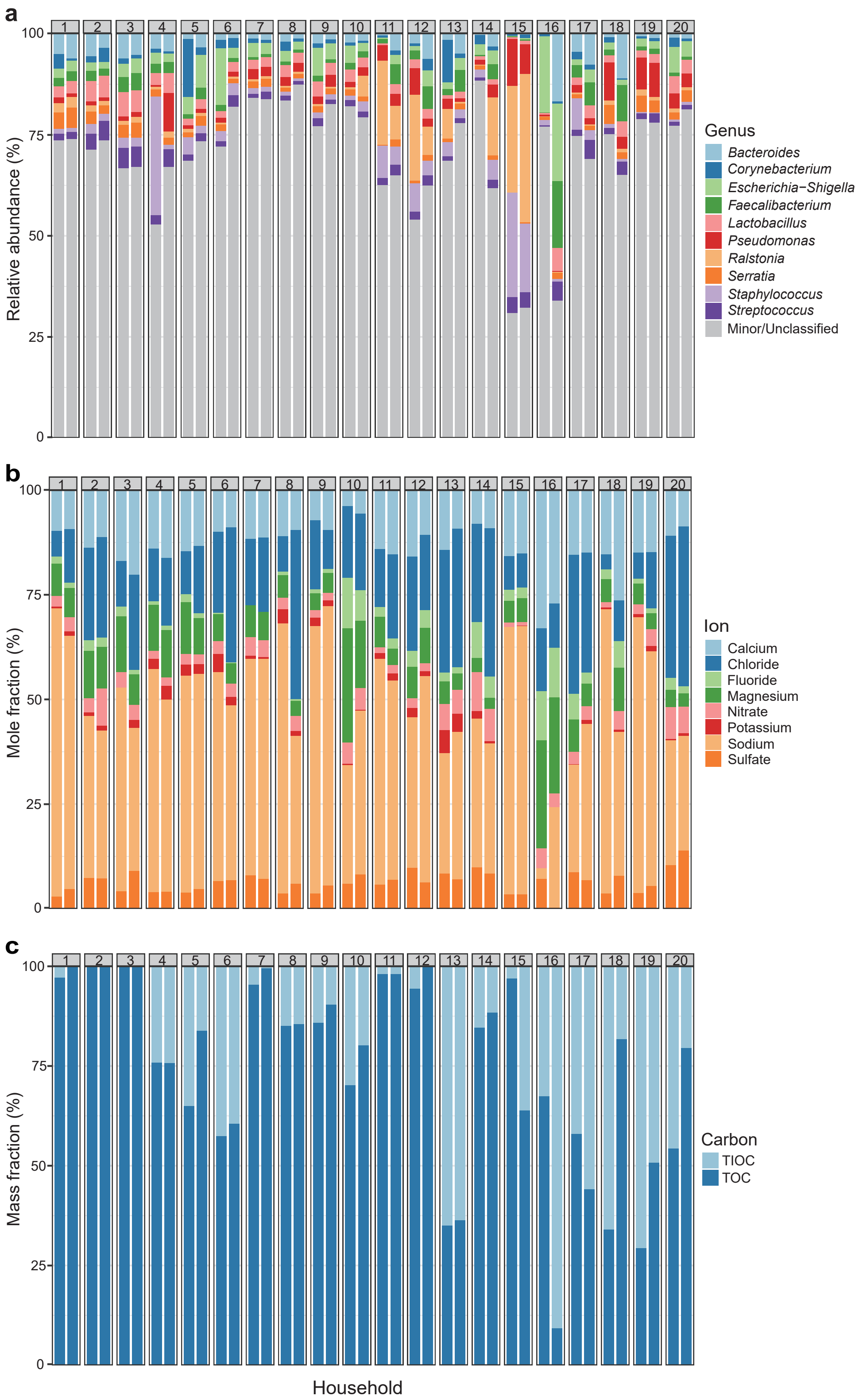

Figure S6. Compositions of biological and chemical constituents on household surfaces on days 5 and 30 from the first sampling round. (a, b, c) Bar plots displaying **(a)** the average relative abundance of the top 10 most abundant genera, **(b)** the average relative mole fraction of detected ions, and **(c)** the average mass fraction of total organic and inorganic carbon across all sampled rooms. In each panel, the left and right bars show day 5 and day 30 compositions, respectively, averaged across all sampled surfaces within each household.

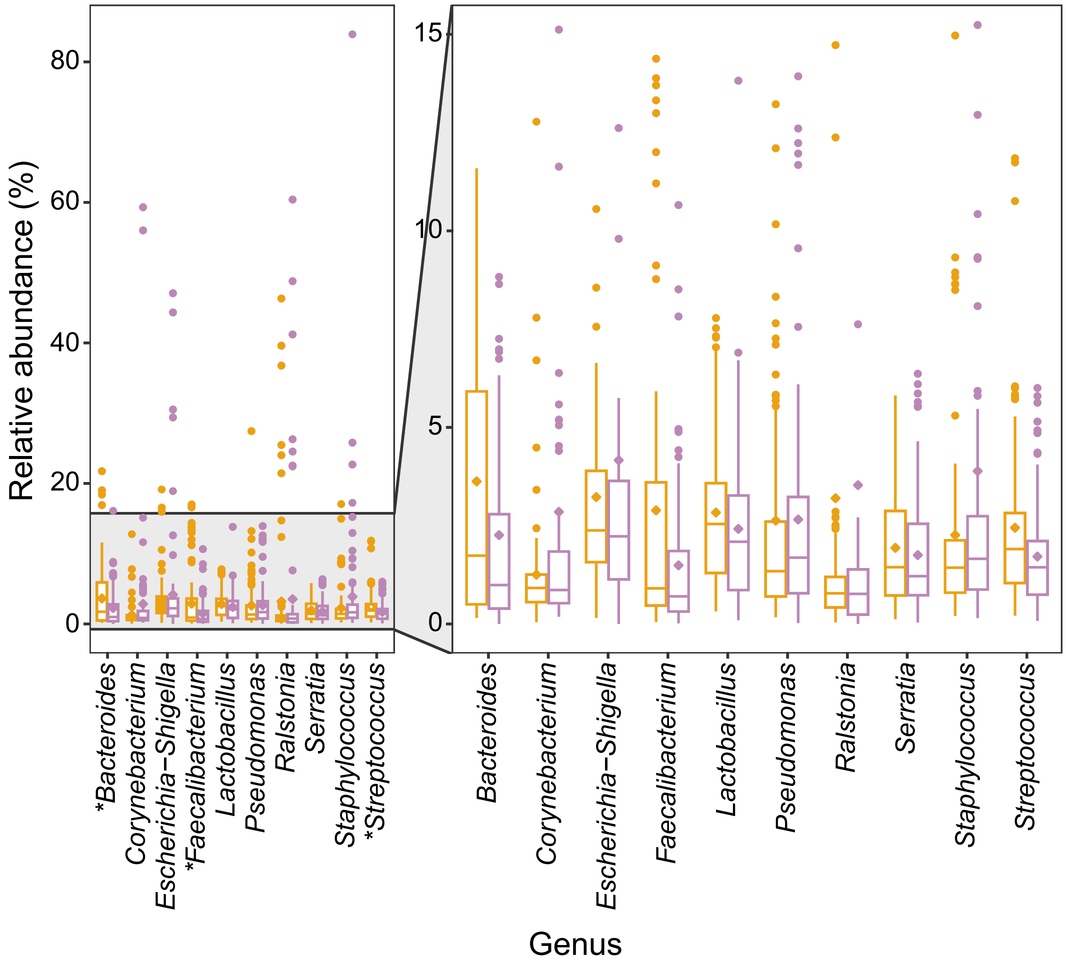

Figure S7. Comparison of the top 10 most abundant genera on household surfaces between days 5 and 30 of the first sampling round, with relative abundances illustrated using boxplots across all sampled household surfaces. A zoomed-in panel highlights data in the lower range. Each box represents the mean (diamond), median, first and third quartiles, and outliers (points beyond the whiskers), with the whiskers extending to 1.5 times the interquartile range. Statistical differences between days 5 and 30 were assessed using the paired Wilcoxon test (****$p < 0.0001$; ***$p < 0.001$; **$p < 0.01$; *$p < 0.05$).

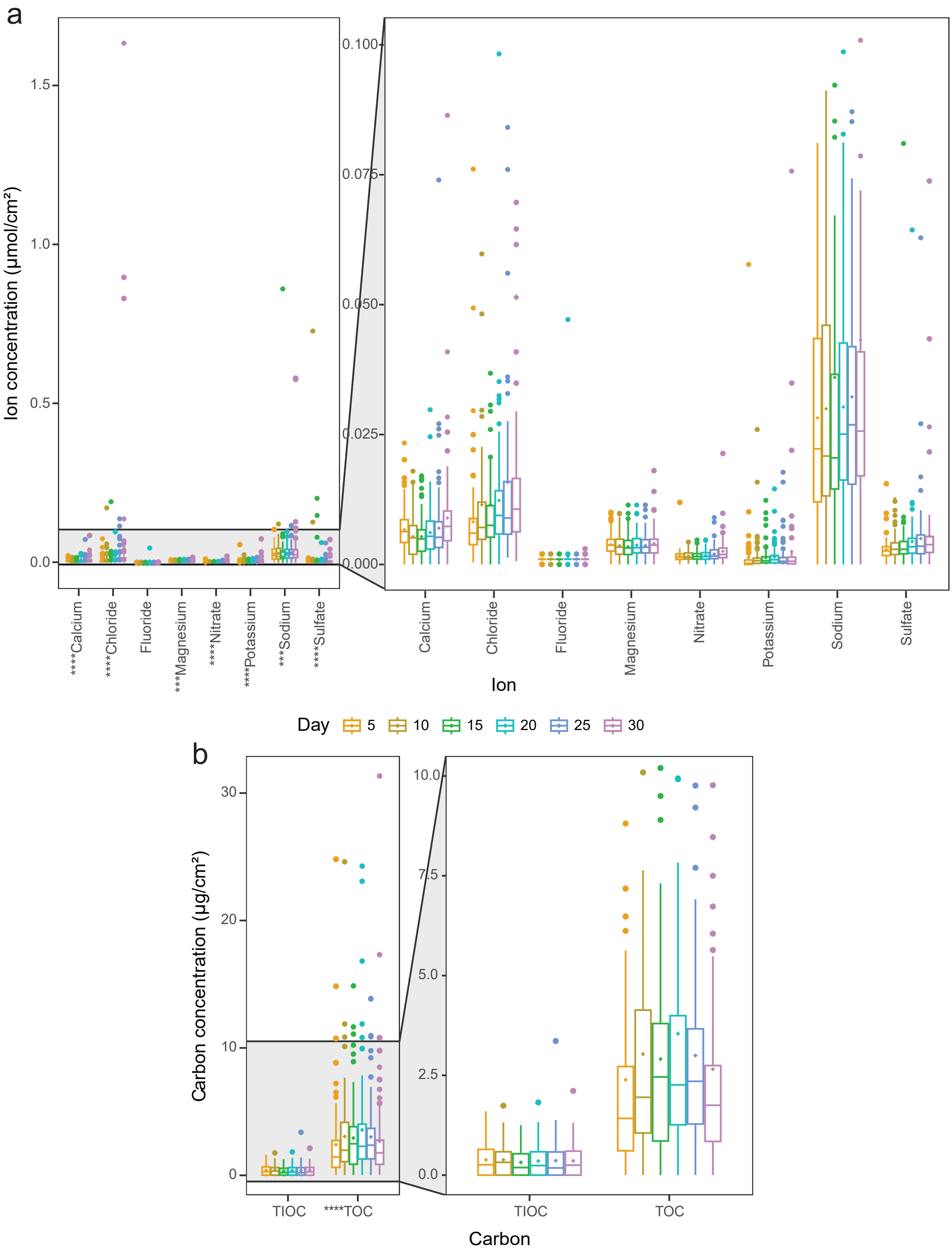

Figure S8. Temporal trends in surface chemical constituents from the first sampling round. (a) Concentrations of detected ions and **(b)** total organic and inorganic carbon measured across all sampled household surfaces from day 5 to day 30. A zoomed-in panel highlights data in the lower range. Each box represents the mean (diamond), median, first and third quartiles, and outliers (points beyond the whiskers), with the whiskers extending to 1.5 times the interquartile range. Temporal changes were assessed using the paired Friedman test (****p < 0.0001; ***p < 0.001; **p < 0.01; *p < 0.05).

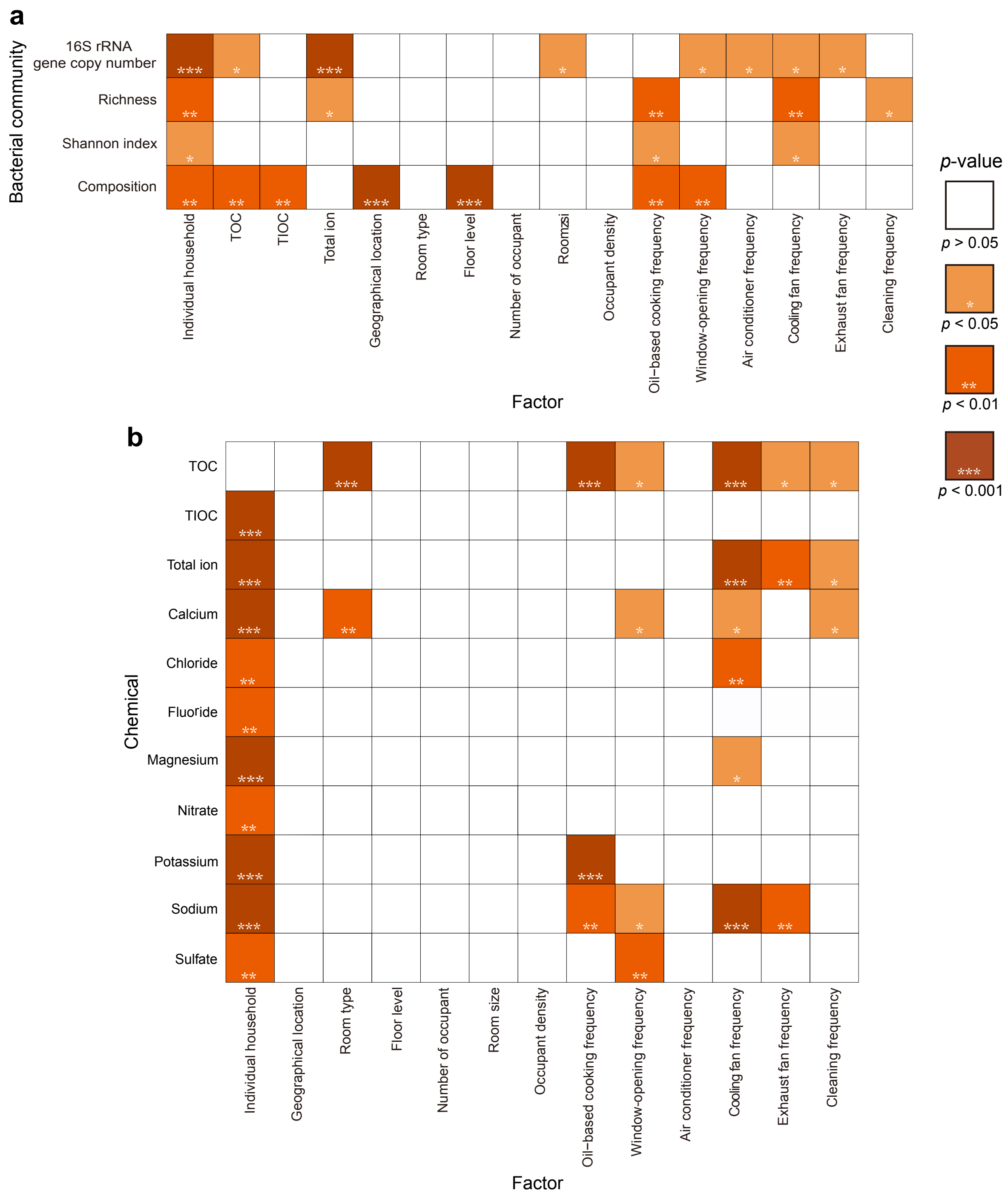

Figure S9. Influences of various factors on bacterial communities and chemical concentrations on household surfaces during the first sampling round. (a) Wald test results from linear mixed-effects models showing the impact of different factors on 16S rRNA gene copy numbers and α-diversity metrics. PERMANOVA was used to assess changes in bacterial community composition based on Bray–Curtis dissimilarity. **(b)** Wald test results showing the impact of different factors on total organic and inorganic carbon concentrations, total ion concentrations, and individual ion concentrations across all sampled household surfaces.

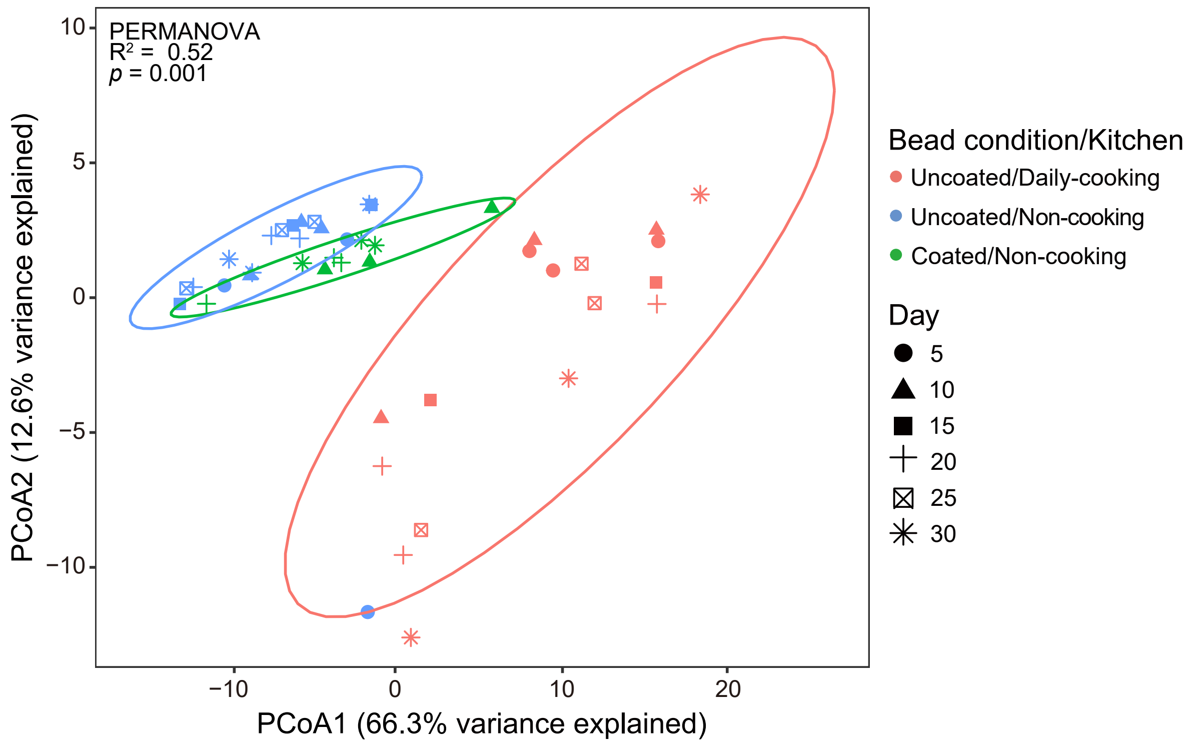

Figure S10. Principal coordinates analysis (PCoA) plot of organic compound compositions on kitchen surfaces from the second sampling round. PERMANOVA was used to assess the Euclidean distances in compound compositions among the three groups of kitchens with varying cooking frequencies and bead conditions. Colored ellipses represent the 95% confidence intervals based on a multivariate t-distribution.

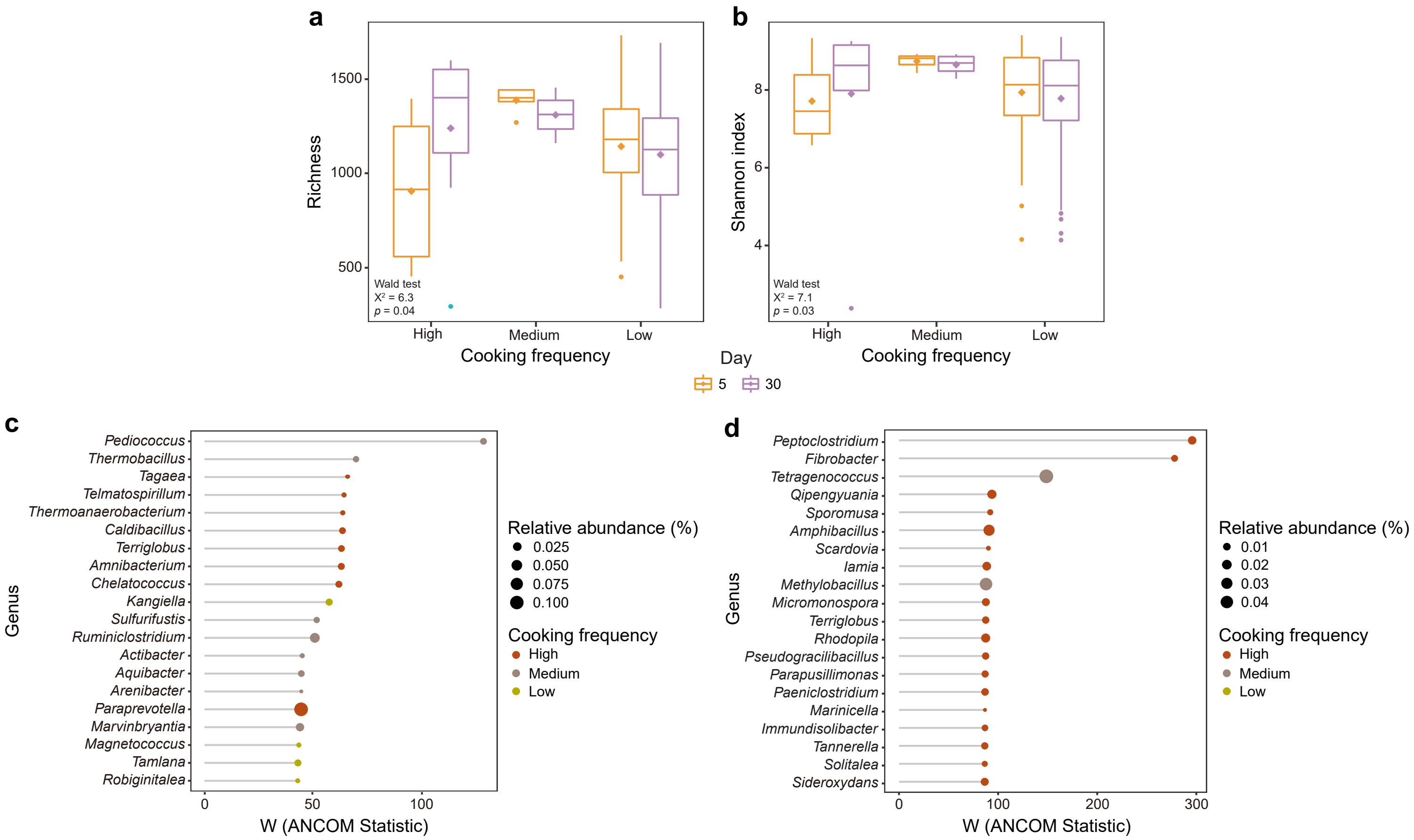

Figure S11. Bacterial diversity and composition on household surfaces from the first sampling round. (a, b) Boxplots illustrating the α-diversity of bacterial communities based on cooking frequencies, measured using **(a)** richness and **(b)** the Shannon index. Each box represents the mean (diamond), median, and first and third quartiles, with the whiskers extending to 1.5 times the interquartile range. Statistical differences over time among the three groups of kitchens with varying cooking frequencies were assessed using the Wald test. **(c, d)** Top 20 differentially abundant genera identified across the three groups on **(c)** day 5 and **(d)** day 30 using an ANCOM-BC. Circle size represents the average relative abundance of each genus within its respective group. The x-axis (W statistic) indicates the magnitude of differential abundance, with larger absolute values representing stronger differences between groups.

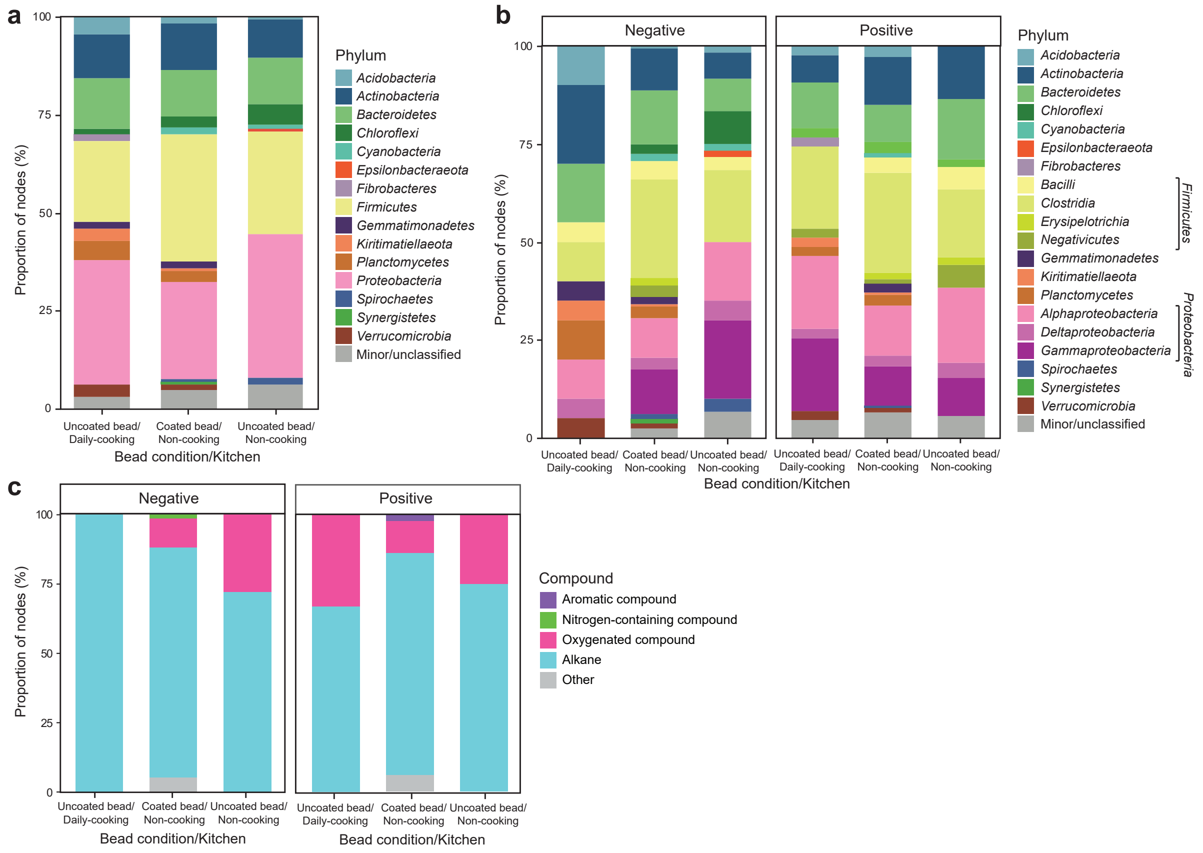

Figure S12. Representation of phyla and organic compounds in the networks of amplicon sequence variants (ASVs) and compounds on kitchen surfaces from the second sampling round. (a) Proportions of nodes representing the top 20 most abundant phyla across the three groups of kitchens with varying cooking frequencies and bead conditions. **(b)** Proportions of these phyla nodes involved in positive or negative interactions with the organic compounds. **(c)** Proportions of compound nodes involved in positive or negative interactions with the ASVs.

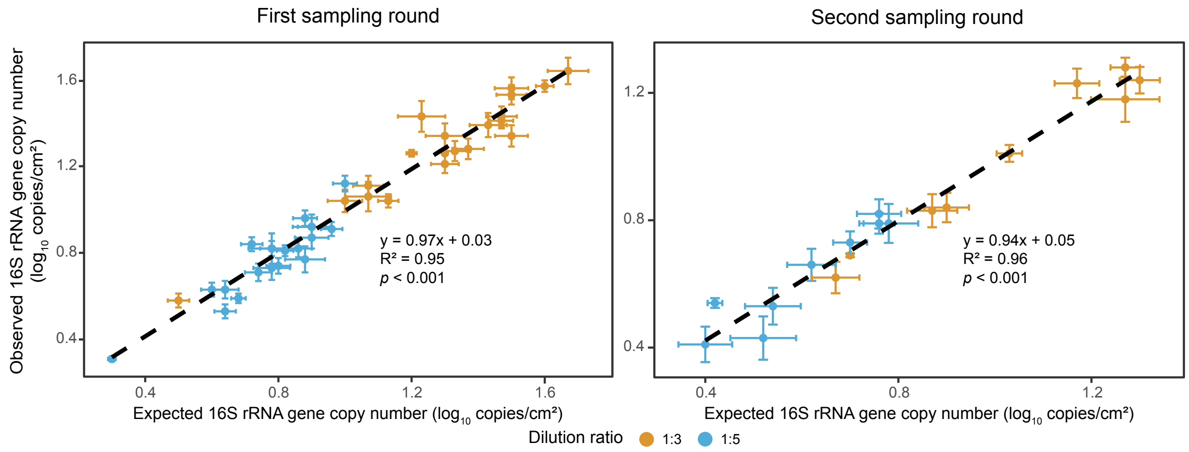

Figure S13. Assessment of PCR inhibition using serial dilutions. Day-30 samples were diluted 1:3 and 1:5, including randomly selected samples from each household in the first sampling round and all kitchen samples in the second. The scatter plot shows expected versus observed 16S rRNA gene copy numbers, with a dashed line indicating the linear regression fit.

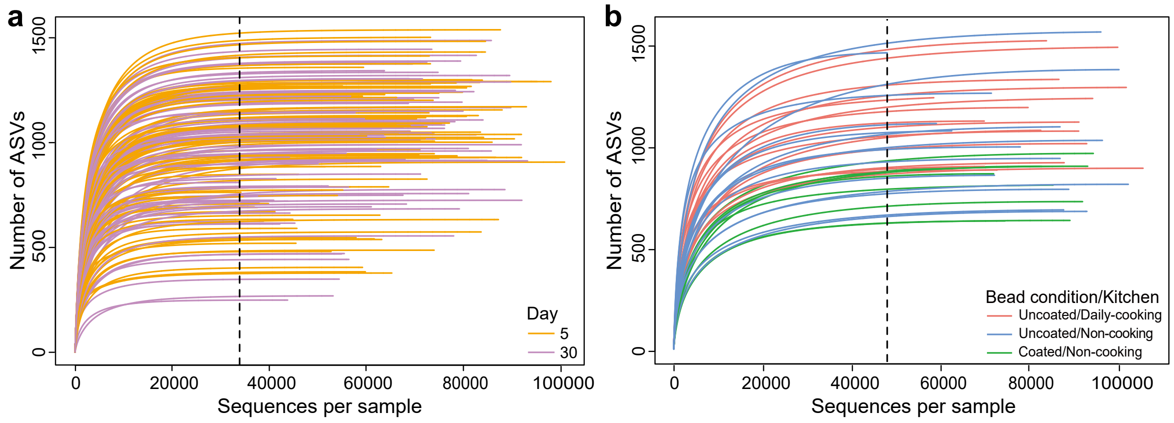

Figure S14. Rarefaction analysis of amplicon sequence variant (ASV) counts. (a, b) Lines representing the number of ASVs identified in samples from the **(a)** first sampling round and **(b)** second sampling round. The vertical dashed line indicates the applied rarefaction depth.

Text S1. Detailed procedures for chemical analysis

Chemical and biological constituents were extracted from glass beads by sonicating them in 2 mL of deionized water for 10 min in an ultrasonic bath (40 kHz; 22 ± 2°C). The extracts were filtered through a 0.22-μm polyethersulfone syringe filter (Biofil, Guangzhou, China). The filters were retained for genomic DNA extraction, while the aqueous extracts were used for chemical analysis.

Total organic carbon (TOC) and total inorganic carbon concentrations were measured using a TOC analyzer (Shimadzu TOC-V CSH; Shimadzu Corp, Kyoto, Japan). Anion (chloride, fluoride, nitrate, and sulfate) concentrations were measured using an ion chromatography system (Dionex ICS-1100; Thermo Fisher Scientific, Waltham, MA, USA) equipped with an IonPac AS18 column, while cation (calcium, magnesium, potassium, sodium, and ammonium) concentrations were measured using an IonPac CS12A column. Both columns were maintained at 30°C. For anion separation, 20 mM potassium hydroxide was used as the eluent, while 31 mM methanesulfonic acid was used for cation separation. The flow rate for both eluents was 1 mL/min.

Organic compounds in the glass bead samples were analyzed using a previously reported procedure[1] with minor modifications. Glass beads from each box were evenly divided into two replicates. Before extraction, 40 ng of deuterated polycyclic aromatic hydrocarbons (PAH-Mix 24 deuterated; LGC Standards GmbH, Wesel, Germany) were added to the samples as internal standards. The samples were extracted using 4 mL of high-performance liquid chromatography-grade dichloromethane (Anaqua, Shanghai, China) for 30 s, followed by three cycles of ultrasonic extraction, each lasting 20 min. The 12 mL combined extract was concentrated to 0.5 mL using a pressurized gas blowing concentrator (LC-DCY-24G; Lichen China Ltd, Shanghai, China) with 99.95% purity nitrogen gas at 35°C. The solution was then centrifuged at 8,000 rpm for 10 min and filtered through a 0.22-μm polyethersulfone syringe

filter (Biofil, Guangzhou, China). Extracts from unused and cleaned glass beads, as well as closed-lid boxes, were used as background references, with their peaks subtracted from the sample extracts.

The extracts were analyzed using gas chromatography–mass spectrometry (Shimadzu QP2010 Ultra; Shimadzu, Kyoto, Japan) in full scan mode, covering a mass range of 50–650 m/z with an electron impact energy of 70 eV. Chromatographic separation was performed in an RTX-5 capillary column (30 m, 0.32 mm I.D., 0.25-μm film thickness; Restek, Bellefonte, PA, USA). The oven temperature was programmed to hold at 60°C for 1 min, then increase to 120°C at 10°C min^{-1}, followed by a ramp to 250°C at 5°C min^{-1} (held for 4 min) and a final rise to 300°C at 15°C min^{-1} (held for 8 min). Ultra-high-purity helium (99.999%) was used as the carrier gas at a flow rate of 1.2 mL/min. Peaks were integrated using Shimadzu PostRun Analysis software (Shimadzu, Kyoto, Japan), and mass spectra were compared against the NIST17 chemical standard library (similarity $\geq$ 90%). The relative abundance of each compound was calculated by dividing its peak area by that of the internal standard. The uniqueness of each compound detected in kitchens with specific oil-based cooking frequencies was assessed using the relative abundance ratio. This ratio was calculated by dividing the compound's relative abundance in kitchens with a specific cooking frequency by its total relative abundance across the three groups of kitchens with varying cooking frequencies and bead conditions in the second sampling round.

Text S2. Detailed procedures for genomic DNA extraction, quantitative polymerase chain reaction (qPCR), and amplicon sequencing

Genomic DNA was extracted from the aforementioned filters using the DNeasy PowerSoil Pro Kit (Qiagen, Germany) following the manufacturer's protocols. Bacterial abundance was quantified by amplifying the 16S rRNA gene with the 515F/806R primer pair.[2,3] qPCR was

performed using a StepOnePlus Real-Time PCR System (Applied Biosystems, Foster City, CA, USA). The thermocycling program began with an initial denaturation at 95°C for 2 min, followed by 40 cycles of denaturation at 95°C for 15 s, annealing at 60°C for 30 s, and extension at 72°C for 30 s. Each 20 µL qPCR reaction consisted of 2 µL of template genomic DNA, 1× PowerTrack SYBR Green Master Mix (Thermo Fisher Scientific, Waltham, MA, USA), and 0.4 mM of each of the forward and reverse primers. Absolute quantification was performed using a standard curve generated from known amounts of *Escherichia coli* BW25113 genomic DNA.[4,5] Standards spanned five orders of magnitude, with an $R^2 > 0.99$ and a PCR efficiency of 98.1% ± 1.3%. Serial dilution tests (1:3 and 1:5) were performed using day-30 samples, randomly selected from each household in the first sampling round and including all kitchens in the second. Expected 16S rRNA gene copy numbers, calculated from dilution factors assuming no PCR inhibition, were compared to observed values measured by qPCR from the diluted samples. Linear regression slopes of 0.96 ($R^2 = 0.95$) and 0.94 ($R^2 = 0.96$) for the first and second sampling rounds, respectively, indicated minimal PCR inhibition (**Fig. S13**). All standards and samples were analyzed in triplicate.

Amplicon sequencing of the 16S rRNA gene was performed using the 515F/806R primer pair[2] following established protocols.[6] Sequencing was conducted on an Illumina MiSeq platform (Novogene Technology Co., Ltd., Beijing, China), generating 250-bp paired-end reads. ZymoBIOMICS microbial community standards (Zymo Research, CA, USA) served as positive controls, while sterile filters processed without samples and beads in closed-lid boxes served as negative controls. The sequence data have been deposited in the Sequence Read Archive of the National Center for Biotechnology Information under accession number PRJNA1191072.

Text S3. Detailed procedures for bioinformatics and statistical analyses

Sequencing reads were processed using the QIIME2 platform (v2021.11)[7] following established protocols.[8,9] Briefly, reads shorter than 240 bp or with an average Phred quality score below 25 were discarded. High-quality reads were denoised, paired-end reads were merged, chimeras were removed, and amplicon sequence variants (ASVs) were generated using the DADA2 plugin[10] in QIIME2. Taxonomic assignment of ASVs was performed using the SILVA 138 reference database, applying a 99% similarity threshold.[11] Potential contaminants were identified in negative controls using the "decontam" package (v1.9.0) in prevalence mode, with a significance threshold of 0.1.[12] Sequences associated with any identified contaminants were removed. After quality filtering, the first sampling round yielded an average of 67,743 ± 15,285 reads and 987 ± 278 ASVs per sample. In the second sampling round, an average of 85,414 ± 13,400 reads and 828 ± 195 ASVs per sample were retained.

All statistical analyses were performed in R (v4.1.2). Statistical comparisons were performed using the "ggpubr" package (v0.6.0), applying paired Wilcoxon tests for two-group comparisons and paired Friedman tests for multiple-group analyses. The relative abundance of ASVs was calculated using the "dplyr" package (v1.0.8). To evaluate the effects of various factors on 16S rRNA gene copy numbers, α-diversity metrics (richness and Shannon diversity index), and chemical concentrations over time, a Bayesian generalized linear mixed-effects model with Markov chain Monte Carlo estimation was applied. Statistical significance was assessed using Wald tests on posterior parameter distributions. All analyses were conducted in R using the "MCMCglmm" package (v2.36). Permutational multivariate analysis of variance was conducted on Bray–Curtis dissimilarities to assess the influences of various factors on bacterial β-diversity and on Euclidean distances to analyze differences in organic compound compositions across the surfaces of kitchens with varying cooking frequencies and bead conditions. Principal coordinates analysis was used to visualize the results for organic

compound compositions. The analyses were performed using the "vegan" package (v2.6-4) using 999 permutations. α-Diversity metrics were calculated after normalizing all samples to the minimum read depth observed in each dataset: 33,900 reads per sample in the first sampling round and 48,700 reads per sample in the second sampling round. Normalization was performed using the "picante" package (v1.8.2). Rarefaction curves, constructed with the "vegan" package (v2.6-4), demonstrated sufficient depth to capture richness across all samples in both sampling rounds (**Fig. S14**).

Differentially abundant genera were identified using the Analysis of Compositions of Microbiomes with Bias Correction (ANCOM-BC) algorithm from the "ancombc" package (v1.4.0). This approach was used to examine differences among the three groups categorized by oil-based cooking frequency in the first sampling round and by cooking frequency combined with bead condition in the second sampling round. Organic compounds associated with compositional differences in bacterial communities among the three groups of kitchens with varying cooking frequencies and bead conditions in the second sampling round were identified using distance-based redundancy analysis with ANOVA ($p < 0.01$). The analysis employed a Bray–Curtis dissimilarity matrix of all ASVs using the "capscale" function in the "vegan" package (v2.6-4). A co-occurrence network analysis was performed on ASVs present in > 30% of samples within each group (stratified by cooking frequency and collection day) from the first sampling round, using the "dplyr" package (v1.0.8). Network connections were established based on Spearman correlation coefficients (ρ), calculated from centered log-ratio (clr)-transformed relative abundances using the "compositions" package (v2.0-6). Separate analyses were conducted for kitchens with low (0–2 days/week) and high (5–7 days/week) cooking frequencies on days 5 and 30. In the second sampling round, correlations between clr-transformed relative abundances of all ASVs and organic compounds were analyzed across all samples collected from days 5 to 30 for the three groups of kitchens with varying cooking

frequencies and bead conditions. Correlations were computed using PyBootNet (100 bootstrap iterations),[13] retaining only strong correlations ($\rho > 0.8$ or $\rho < -0.8$). Network visualization and analysis of parameters such as weighted node degree, modularity, and density were conducted using Gephi (v0.9.6).

References

(1) Styler, S. A.; Baergen, A. M.; Donaldson, D.; Herrmann, H., Organic composition, chemistry, and photochemistry of urban film in Leipzig, Germany. *ACS Earth Space Chem.* **2018,** *2*, (9), 935-945, DOI: 10.1021/acsearthspacechem.8b00087.

(2) Caporaso, J. G.; Lauber, C. L.; Walters, W. A.; Berg-Lyons, D.; Lozupone, C. A.; Turnbaugh, P. J.; Fierer, N.; Knight, R., Global patterns of 16S rRNA diversity at a depth of millions of sequences per sample. *Proc. Natl. Acad. Sci.* **2011,** *108*, (supplement_1), 4516-4522, DOI: 10.1073/pnas.1000080107

(3) Tkacz, A.; Hortala, M.; Poole, P. S., Absolute quantitation of microbiota abundance in environmental samples. *Microbiome* **2018,** *6*, 1-13, DOI: 10.1186/s40168-018-0491-7

(4) Larionov, A.; Krause, A.; Miller, W., A standard curve based method for relative real time PCR data processing. *BMC Bioinf.* **2005,** *6*, (1), 1-16, DOI: 10.1186/1471-2105-6-62

(5) Lee, B. G.; Yang, J. I.; Kim, E.; Geum, S. W.; Park, J. H.; Yeo, M. K., Investigation of bacterial and fungal communities in indoor and outdoor air of elementary school classrooms by 16S rRNA gene and ITS region sequencing. *Indoor Air* **2021,** *31*, (5), 1553-1562, DOI: 10.1111/ina.12825

(6) Caporaso, J. G.; Lauber, C. L.; Walters, W. A.; Berg-Lyons, D.; Huntley, J.; Fierer, N.; Owens, S. M.; Betley, J.; Fraser, L.; Bauer, M., Ultra-high-throughput microbial community analysis on the Illumina HiSeq and MiSeq platforms. *The ISME journal* **2012,** *6*, (8), 1621-1624, DOI: 10.1038/ismej.2012.8

(7) Caporaso, J. G.; Kuczynski, J.; Stombaugh, J.; Bittinger, K.; Bushman, F. D.; Costello, E. K.; Fierer, N.; Peña, A. G.; Goodrich, J. K.; Gordon, J. I., QIIME allows analysis of high-throughput community sequencing data. *Nat. Methods* **2010,** *7*, (5), 335-336, DOI: 10.1038/nmeth.f.303

(8) Yu, J.; Tang, S. N.; Lee, P. K., Microbial communities in full-scale wastewater treatment systems exhibit deterministic assembly processes and functional dependency over time. *Environ. Sci. Technol.* **2021,** *55*, (8), 5312-5323, DOI: 10.1021/acs.est.0c06732

(9) Zhou, Y.; Leung, M. H.; Tong, X.; Lee, J. Y.; Lee, P. K., City-scale meta-analysis of indoor airborne microbiota reveals that taxonomic and functional compositions vary with building types. *Environ. Sci. Technol.* **2021,** *55*, (22), 15051-15062, DOI: 10.1021/acs.est.1c03941

(10) Callahan, B. J.; McMurdie, P. J.; Rosen, M. J.; Han, A. W.; Johnson, A. J. A.; Holmes, S. P., DADA2: High-resolution sample inference from Illumina amplicon data. *Nat. Methods* **2016,** *13*, (7), 581-583, DOI: 10.1038/nmeth.3869

(11) Quast, C.; Pruesse, E.; Yilmaz, P.; Gerken, J.; Schweer, T.; Yarza, P.; Peplies, J.; Glöckner, F. O., The SILVA ribosomal RNA gene database project: improved data processing and web-based tools. *Nucleic Acids Res.* **2012,** *41*, (D1), D590-D596, DOI: 10.1093/nar/gks1219

(12) Davis, N. M.; Proctor, D. M.; Holmes, S. P.; Relman, D. A.; Callahan, B. J., Simple statistical identification and removal of contaminant sequences in marker-gene and metagenomics data. *Microbiome* **2018,** *6*, 1-14, DOI: 10.1093/nar/gks1219

(13) Akhavan, S. R.; Kelley, S. T., PyBootNet: a python package for bootstrapping and network construction. *PeerJ* **2025,** *13*, e18915, DOI: 10.7717/peerj.18915
